# Baseline multi‐omics signatures could predict therapeutic response to neoadjuvant anti‐PD‐1 immunochemotherapy in non‐small‐cell lung cancer

**DOI:** 10.1002/ctm2.70579

**Published:** 2026-01-07

**Authors:** Ailing Cao, Yaobin Lin, Shaoxing Guan, Youhao Chen, Wenyu Zhai, Yuheng Zhou, Shoucheng Feng, Yanping Guan, Yiyu Zhang, Min Huang, Xueding Wang, Hao Long

**Affiliations:** ^1^ Institute of Clinical Pharmacology School of Pharmaceutical Sciences Sun Yat‐Sen University Guangzhou China; ^2^ Department of Thoracic Surgery State Key Laboratory of Oncology in South China Collaborative Innovation Center for Cancer Medicine Sun Yat‐sen University Cancer Center Guangzhou China; ^3^ Lung Cancer Research Center Sun Yat‐Sen University Guangzhou China

**Keywords:** gut microbiota, metabolomics, neoadjuvant, NSCLC, PD‐1

## Abstract

**Background:**

Neoadjuvant anti‐programmed cell death 1 (PD‐1) immunochemotherapy has shown promising efficiency in the treatment of early‐stage non‐small‐cell lung cancer (NSCLC), but it has not consistently yielded durable responses. Biomarkers for the prediction of efficacy are warranted.

**Methods:**

We performed shotgun metagenomic and plasma/faecal metabolomic studies in 44 NSCLC patients who underwent neoadjuvant tislelizumab plus platinum‐based doublet chemotherapy. Samples were collected at baseline and before surgical resection, and the major pathologic response (MPR) was evaluated.

**Results:**

MPR patients showed a significantly higher gut‐microbial alpha diversity, an enrichment of *Ruminococcaceae*, *Lachnospiraceae* and *Clostridiales* species, and an increased plasma level of tryptophan metabolites at baseline. On the contrary, non‐MPR patients were characterized by enrichment of *Prevotella* species in faecal samples and higher plasma levels of linoleic acid metabolites. A high predictive accuracy was achieved using a small panel of differential microbial (*Clostridium sp. M62/1* and *Eisenbergiella tayi*) or metabolomic features (linoleic acid, oxindole‐3‐acetic acid and quinolinic acid) with AUCs > .85.

**Conclusions:**

The baseline characteristics of the gut microbiota and plasma metabolites could provide early predictions of the response to neoadjuvant anti‐PD‐1 immunochemotherapy.

**Trial registration:**

NCT05244837.

**Key points:**

- Baseline metagenomic and metabolomic signatures were significantly associated with the major pathologic response of neoadjuvant anti‐PD‐1 immunochemotherapy.
- Integrated microbial model (consists of *Clostridium* sp. *M62/1* and *Eisenbergiella tayi*) and metabolomic model (consists of linoleic acid, oxindole‐3‐acetic acid and quinolinic acid) could provide early predictions of the response.

## Graphical abstract

Overview of study design. Metagenomic and plasma/fecal metabolomic studies were performed in 44 NSCLC patients who underwent neoadjuvant tislelizumab therapy plus platinum ‐ based doublet chemotherapy.

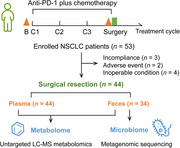

## 1 BACKGROUND

Non‐small‐cell lung cancer (NSCLC), the most prevalent type of lung cancer, is the primary driver of cancer‐related death worldwide. NSCLCs are relatively insensitive to chemotherapy. Immune checkpoint inhibitors (ICIs) that target the PD‐1/PD‐L1 pathway have revolutionized the treatment of malignancies, including NSCLC. Since 2015, the FDA has approved the first ICI, nivolumab, for advanced NSCLC following the failure of platinum‐based chemotherapy. Since 2020, neoadjuvant ICIs have been increasingly incorporated into the perioperative treatment of resectable NSCLC. The integration of immunotherapy in early‐stage NSCLC has shown promising outcomes. However, only 30%‐56% of patients with resectable NSCLC achieved major pathologic response from this treatment,^1^, ^2^, ^3^ whereas up to 32%‐63% experience grade 3+ treatment‐related adverse events.^1^ In this context, reliable biomarkers are urgently needed for neoadjuvant ICIs therapy.

To date, PD‐L1 expression serves as the most widely accepted biomarker for predicting the efficacy of ICIs. However, PD‐L1 expression has not been found to be correlated with the pathological response to neoadjuvant anti‐PD‐1 therapy in resectable NSCLC, although it is associated with the response in advanced NSCLC.^4^, ^5^ So far, other biomarkers, including nonsynonymous mutations, high tumour mutation burden, neoantigens, high microsatellite instability and microbiota, are also considered potential predictors of responses to ICI treatment.^6^ Among these, gut microbial characteristics hold significant importance.

Several studies have demonstrated a strong association between gut microbiome (e.g., *Akkermansia* and *Bifidobacterium*) and the anti‐PD‐1 immunotherapy response in many types of malignancies, including advanced NSCLC.^7^, ^8^ These findings provide important insights for predicting the anti‐PD‐1 immunotherapy efficacy in such diseases.^9^ However, no study has been conducted in patients with resectable NSCLC undergoing neoadjuvant immunochemotherapy. Given that the predictive value of biomarkers can vary depending on cancer types and stages, as exemplified by PD‐1 expression levels mentioned previously, investigating microbial profiles and underlying mechanisms in early‐stage NSCLC treated with neoadjuvant immunochemotherapy could yield significant clinical implications.

Meanwhile, metabolomic studies have recently been applied to identify biomarkers predicting drug responses. Some studies have shown that early on‐treatment levels of histidine, lactate and hypoxanthine are associated with the response of anti‐PD‐1 monotherapy,^10^, ^11^ and baseline N‐(3‐Indolylacetyl)‐L‐alanine was identified as a predictor of the outcome in patients who underwent PD‐1 inhibitors plus chemotherapy.^12^ Additionally, researchers have also considered that the impact of gut microbiota on the efficacy of ICIs is mediated by gut microbiota‐derived metabolites, such as short‐chain fatty acids and bile acids. These metabolites enter the systemic circulation, influencing immune cells, regulating host immunity, and modulating drug responses.^13^ Moreover, significant changes in the metabolomic profile have been observed as NSCLC progresses.^14^

Therefore, we conducted the metagenomic and untargeted plasma/faecal metabolomic study in NSCLC patients receiving neoadjuvant anti‐PD‐1 immunochemotherapy. In this population, we studied the signatures of gut microbiota and plasma/faecal metabolomic profiles, and their associations with the pathological response. Specifically, the microbe‐metabolite interactions relevant to the treatment outcome were investigated.

## 2 METHODS

### 2.1 Study population and sample collection

This was a subgroup analysis of LungMark research, a phase 2 prospective clinical trial to explore the efficacy of biomarkers of neoadjuvant anti‐PD‐1 immunochemotherapy in NSCLC. Patients were recruited from Sun Yat‐sen University Cancer Center between December 2020 and November 2022. The trial was approved by the Ethics Committee and Institutional Review Board of Sun Yat‐sen University Cancer Center (A2020‐008) and registered on ClinicalTrials.gov (NCT05244837). Written informed consent was obtained from all subjects. The main inclusion criteria include: (1) histologically confirmed stage IIB‐IIIA NSCLC, (2) Eastern Cooperative Oncology Group (ECOG) performance status of 0 or 1, (3) eligibility for R0 resection. The main exclusion criteria were as follows: (1) received other systemic anticancer therapy or immunomodulators 4 weeks before the first dose, (2) a serious infection occurred or received antibiotics within 14 days before enrollment. The full details of the study design and the inclusion/exclusion criteria were provided in the Supplementary Information for Study Protocol.

Participants received standard neoadjuvant treatment with platinum‐based doublet chemotherapy plus anti‐PD‐1 immunotherapy (Tislelizumab) every three weeks for 3–4 cycles before surgical resection. For squamous cancer, carboplatin plus paclitaxel/nab‐paclitaxel was used in chemotherapy. For non‐squamous cancer, carboplatin plus pemetrexed was used. Demographic information, diagnosis and biopsy biomarkers were retrieved from medical records. The major pathologic response (MPR), defined as less than 10% viable tumour cells in the pathologically resected specimen, was evaluated.^15^ Pathologic complete response (pCR), disease‐free survival (DFS) and radiographical response according to the RECIST v 1.1 criteria were also assessed.

Faecal and plasma samples were collected at baseline and before surgical resection. Patients were instructed to collect their stools using a plastic emesis basin and a 15 mL sterile stool collector containing Swab DNA locker (80802‐100, TIANDZ, China). Faecal samples were kept at room temperature temporarily and brought to the laboratory within 24 h after collection, where all samples were homogenized and finally stored at −80°C. Venous blood was collected and centrifuged at 4000 rpm for 10 min to obtain the fresh plasma before storing at −80°C until analysis. Paired samples collected at both baseline and before surgical resection were included in the subsequent microbial feature profiling.

### 2.2 Metagenomic profiling

Metagenomic DNA was extracted from faecal samples using the Magnetic Stool DNA Extraction Kit (AU46111‐96, BioTeke, China). The DNA concentrations were quantified using the dsDNA Assay Kits (Q33230, Invitrogen, USA). A total of 200 ng DNA was fragmented by the Bioruptor Pico (Diagenode, Belgium). The length of DNA fragments was quantified using the Agilent 2100 Bioanalyzer. Samples were sequenced (paired‐end, 150 bp) on the NovaSeq 6000 platform (Illumina, USA) with a NovaSeq 6000 XP4‐LaneKitv1.5 Reagent Kits (300 cycles) (20043131, Illumina, USA) at the LC‐Bio Technologies. A negative extraction control (using nuclease‐free water instead of the sample) was also processed along with the faecal samples.

The sequencing adapters were first removed using Cutadapt v1.9, followed by quality trimming of low‐quality reads using fqtrim v0.94. After aligning reads to the host genome and filtering out human host DNA contamination using the bowtie2 v2.2.0, the quality‐filtered reads were de novo assembled to construct the contigs by MEGAHIT v1.2.9. All coding regions were predicted by MetaGeneMark v3.25 and clustered by CD‐HIT v4.6.1 to obtain Unigenes. The abundance of Unigenes was estimated based on aligned read numbers using the bowtie2 v2.2.0. The taxonomy was obtained by aligning Unigenes against the NCBI database using the DIAMOND v 0.9.14. Similarly, the Kyoto Encyclopedia of Genes and Genomes (KEGG) orthology (KO) annotation and the Virulence Factor Database (VFDB) annotation of Unigenes were obtained for subsequent statistical analysis. The quality statistics of sequencing were listed in Table S1.

### 2.3 Metabolomic profiling

For plasma sample preparation, 50 µL of the sample was added with 200 µL of pre‐cooled acetonitrile, and then thoroughly homogenized for 1 min and placed at 4°C for 5 min. After centrifugation at 15000 rpm and 4°C for 10 min, 200 µL of supernatant was concentrated to dryness by vacuum. The lyophilized sample was stored at −80°C until detection (within 1 month).

For faecal sample preparation, 50 mg of freeze‐dried sample was completely homogenized with 250 µL of ice‐cold distilled water. Then, 500 µL of pre‐cooled methanol was added to the tube for extraction. The mixture was completely mixed for 3 min and placed at 4°C for 30 min, and subsequently centrifuged at 15000 rpm, 4°C for 20 min. To remove the Swab DNA locker and enrich metabolites, the supernatant (500 µL) was transferred into the Oasis MAX Cartridge (186004649, Waters, USA) for solid phase extraction. After washing the column with distilled water, the metabolites were eluted by 200 µL of methanol, lyophilized and stored at −80°C until detection (within 1 month).

Meanwhile, quality control (QC) samples, pooling an equal aliquot of each plasma/faecal sample, were parallelly prepared as described above. QC samples were inserted before and after every 8 samples, and 24 samples were regarded as a batch. On the day of starting a batch detection, lyophilised samples were reconstituted in 100 µL (plasma sample) or 150 µL (faecal sample) of methanol/water (1:9), vortexed for 1 min, placed at 4°C for 5 min and subsequently centrifuged for 10 min at 15 000 rpm and 4°C. The supernatant was transferred into the vial and placed in the autosampler operating at 8°C for LC‐MS detection.

Metabolic profiles were generated based on Ultimate 3000 UHPLC coupled with Q‐Exactive Orbitrap mass spectrometer (Thermo Scientific, USA). ACQUITY UPLC HSS T3 Column (100 mm × 2.1 mm, 1.8 µm; Waters) was used for the chromatographic separation. For positive ion mode analysis, the mobile phase consisted of water (A) and acetonitrile (D), both containing .1% formic acid. The following elution gradient was applied: 0–1 min, 5% D; 1–22 min, 5–100% D; 22–26 min, 100% D; 26‐26.1 min, 100–5% D; 26.1‐30 min, 5% D. For negative ion mode, the mobile phases B and C were water/methanol (5:95, v/v) and water with 6.5 mM ammonium formate. The elution gradient was set as follows: 0–1 min, 2% B; 1–18 min, 2–100% B; 18–22 min, 100% B; 22–22.1 min, 100–2% B; 22.1–25 min, 2% B. The column temperature was set at 50°C and 55°C for the positive and negative modes, respectively. For both ion modes, a 5 µL sample was injected and eluted in .35 mL/min. Before and after injection, the needle was mashed with 200 µL 50% methanol/ water for three times.

The mass spectrometer was operated with capillary and aux gas temperatures of 325°C and 350°C, respectively. The scan range was 70–1050 m/z in a resolution of 70 000 for full scan and 17 500 for data‐dependent MS/MS (ddMS^2^) scan in both modes. The top 10 intense ions in a cycle of full scan were filtered for fragmentation. Ion was fragmented at the normalized energy of 15, 30, and 45. The mass spectrometer was tuned before use.

Raw data was analyzed by MS‐DIAL.^16^ The MSP spectral database used for annotation was ESI(±)‐MS/MS from an authentic standards library (version 17) provided by MS‐DIAL. Peak areas were corrected by the systematic error removal using the random forest method (SERRF)^17^ for subsequent statistical analysis. The quality statistics of metabolomic detection were illustrated in Figure S1.

## 3 STATISTICAL ANALYSIS

### 3.1 Overall composition of microbiota and metabolites

Metagenomic data cleaning was first performed by dropping the rare species with average relative abundance <.01% and represented within 10% of the study subjects. This process limited the number of annotations to 499 species and 300 KO functions. The alpha‐diversity (using the Simpson and Shannon indices) was calculated by the vegan package in R.^18^ Taking the compositional feature of microbiome sequencing data into consideration, the beta‐diversity was calculated based on Aitchison distance^19^, ^20^ using the robCompositions package in R.^21^ PERMANOVA was used for distance‐based hypothesis testing.

Metabolomic data cleaning was performed by removing the redundant adducts that were within an m/z window of .0001. Metabolites present within 30% of the study subjects were also dropped. Finally, 1498 plasma metabolites and 2743 faecal metabolites remained for further analysis. PCoA analysis of metabolites was performed based on Bray–Curtis distance with PERMANOVA test.

### 3.2 Differential feature discovery

The important differential features (species, KO functions or metabolites) associated with patient response were investigated using three approaches: (1) Univariate analysis, Welch's T test was used for normally distributed data, while the Wilcoxon Mann–Whitney test was used for non‐normal data with rstatix package in R. (2) Multivariate linear model, R package of MaAsLin2^22^ was used. Age and tumour histology were adjusted in the reduced model. In the full model, sex, weight and smoking history were further adjusted. (3) Random forest model, the R package of randomForest was used to build the random forest model. Clinical metadata of demographic information and tumour diagnosis were also included in model development. Samples were randomly split into two subsets for training (70%) and validation (30%). Parameters including the number of trees, maximum features and maximum depth were selected using the random search algorithm with 10‐fold repeated cross‐validation. The feature importance was measured by the mean decrease in accuracy (MDA). Taking the compositional feature of microbiome sequencing data into consideration, a compositional data analysis (CoDa) method of ALDEx2 was also used in the non‐rarefied dataset to validate the differential species.^23^, ^24^ Microbial sequencing read counts were transformed using the centred log‐ratio (CLR) method, while metabolite peak areas were normalized and Pareto‐scaled. The *p*‐values of all statistical analyses were also adjusted by the Benjamini–Hochberg (BH) method to obtain the FDR value.

### 3.3 Microbe–metabolite association

Spearman's rank correlation analysis was conducted using the pairwise data from all sampling times, which included all differential species (full MaAsLin model *p *< .05) and pathway‐enriched metabolites. To assess the distance‐based relationships, a distance correlation (dCor) analysis was further applied using the HAllA tool.^25^ The *p*‐values of all analyses were also adjusted by the BH method to obtain the FDR value.

### 3.4 Prediction model

Logistic regression models, including the important differential features, were built using the glm function in R. The stepwise method was used to optimize the integrated model. ROC curve and calibration curve were used to assess and visualize the predictive accuracy of the model. Goodness‐of‐fit of the model was also assessed using the Hosmer–Lemeshow (HL) test. Bootstrap resampling analysis with 1000 replications was conducted to calculate the 95% confidence interval (CI) of sensitivity, specificity, accuracy and area under the curve (AUC) of the ROC curve. The final models were further evaluated using a 10‐fold cross‐validation method. Moreover, decision curve analysis was performed using the rmda package to quantify the net benefits. Cox proportional hazard regression models were also built based on the selected differential features. The prediction accuracy for the DFS was assessed using the time‐dependent ROC curve analysis.

## 4 RESULTS

### 4.1 Patient characteristics

Fifty‐three patients were enrolled in this study. Among them, three patients refused to continue the therapy or surgery due to personal reasons; two patients discontinued from therapy due to grade 3–4 adverse events; four patients were determined ineligible for surgical resection for curative intent (Graph Abstract). The remaining 44 patients completing the neoadjuvant combined anti‐PD‐1 therapy and surgical resection were included for further analysis. Patient characteristics, blood chemistry and haematology of study populations were shown in Table 1 and Table S2, respectively. None of the 44 patients received antibiotics, PPIs or probiotics during the neoadjuvant therapy. Thirty‐two (73%) and twenty‐one (48%) patients achieved MPR and pCR, respectively. Compared with adenocarcinoma, patients with squamous NSCLC had a higher rate of MPR (Table 1). With the median follow‐up of 24.5 months, the 2‐year DFS rate was 78%, which was significantly higher among MPR or pCR patients (Figure S2). The radiographical response, which was thought to be limited in assessing the response to neoadjuvant immunotherapy,^4^, ^26^ was significantly associated with MPR in this study (Table 1). It is consistent with other neoadjuvant immunochemotherapy studies and underlines the inherent differences between monotherapy and combined therapy.^5^ Previously reported biomarkers of immunotherapy response, including the PD‐L1 expression, TMB and baseline neutrophil‐to‐lymphocyte ratio,^6^, ^27^ were also evaluated and found uncorrelated with the pathologic response. Age was imbalanced in patients with MPR and the non‐MPR group, which was therefore adjusted in the discovery of differential features along with tumour histology.

**TABLE 1:** Clinical characteristics of study populations (*n *= 44).

Characteristics	Total (*n *= 44)	MPR (*n *= 32)	non‐MPR (*n *= 12)	*p*‐value^a^
Age, years (median, [IQR])	61 [56–66]	63 [58–67]	56 [48–64]	**.026**
Height, cm (median, [IQR])	168 [164–172]	167 [165–172]	168 [158–173]	.969
Weight, kg (median, [IQR])	62.5 [55.3–70.9]	60.0 [53.9–69.6]	67.9 [58.8–72.4]	.131
Sex (*n* [%])				.141
Male	37 [84.1]	29 [90.6]	8 [66.7]	
Female	7 [15.9]	3 [9.4]	4 [33.3]	
Smoking history (*n* [%])				.090
Yes	32 [72.7]	26 [81.2]	6 [50.0]	
No	12 [27.3]	6 [18.8]	6 [50.0]	
Stage (*n* [%])				.192
II	7 [15.9]	7 [21.9]	0 [0]	
III	37 [84.1]	25 [78.1]	12 [100.0]	
Histology (*n* [%])				**.001**
Adenocarcinoma	10 [22.7]	3 [9.4]	7 [58.3]	
Squamous carcinoma	30 [68.2]	27 [84.4]	3^25^	
Others	4 [9.1]	2 [6.2]	2 [16.7]	
Pathological assessment (*n* [%])				**<.001**
pCR	21 [47.7]	21 [65.6]	0 [0]	
non‐pCR	23 [52.3]	11 [34.4]	12 [100]	
RECIST (*n* [%])				**.001**
CR/PR	37 [84.1]	31 [96.9]	6 [50.0]	
SD	5 [11.4]	1 [3.1]	4 [33.3]	
PD	2 [4.5]	0 [0]	2 [16.7]	
PD‐L1 expression (*n* [%])				.304
TPS < 50%	10 [22.7]	5 [15.6]	5 [41.7]	
TPS ≥ 50%	7 [15.9]	6 [18.8]	1 [8.3]	
Unknown	27 [61.4]	21 [65.6]	6 [50.0]	
TMB (*n* [%])				.109
<10 mutation/Mb	15 [34.1]	7 [21.9]	8 [66.7]	
≥10 mutation/Mb	11 [25.0]	9 [28.1]	2 [16.7]	
Unknown	18 [40.9]	16 [50.0]	2 [16.7]	
NLR (median, [IQR])	2.7 [1.7–3.6]	2.7 [1.5–3.6]	2.6 [2.0–3.7]	.524

Abbreviations: CR, complete response; NLR, baseline neutrophil‐to‐lymphocyte ratio; pCR, pathologic complete response; PD, progressive disease; PR, partial response; SD, stable disease; TMB, tumour mutation burden at baseline; TPS, tumour cell proportion score of PD‐L1 at baseline.

^a^ Wilcoxon Mann–Whitney test was used for continuous variables; continuity‐corrected Pearson's Chi‐Square test was used for categorical variables.

### 4.2 Baseline microbial profiles are associated with the major pathologic response of neoadjuvant anti‐PD‐1 immunochemotherapy

**FIGURE 1:**
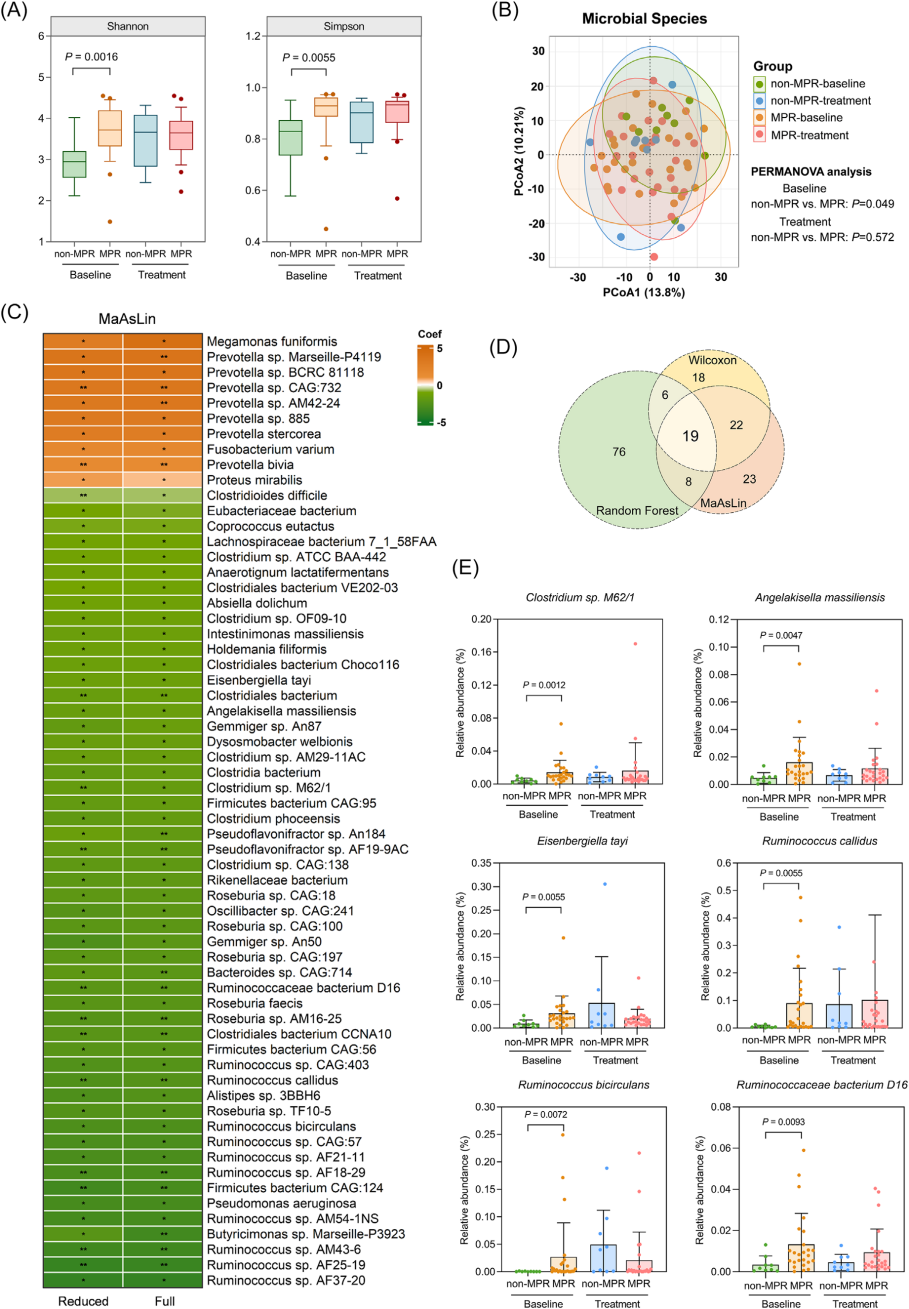
Compositional differences of gut microbiome are associated with the major pathologic response of neoadjuvant anti‐PD‐1 immunochemotherapy. (A) The indices of alpha‐diversity in each group. Shannon and Simpson values were compared using the Wilcoxon Mann–Whitney test. (B) The beta‐diversity showing the overall compositional differences between groups. Principal coordinate analysis (PCoA) was based on Aitchison distance and tested by permutational multivariate analysis of variance (PERMANOVA). (C) The β coefficient of the multivariate linear model showing the species that are significantly associated with the pathologic response. Orange squares indicate unfavourable enrichment in the non‐MPR group, while green squares indicate favourable enrichment in the MPR group. Age and tumour histology were adjusted in the reduced model. Sex, weight and smoking history were further adjusted in the full model. Symbols: *** *p* < .001; ** *p* < .01; *p < .05. (D) The number of differential species tested by three statistical methods. A total of 65 and 72 species achieved a *p*‐value of < .05 in univariate analysis and multivariate linear analysis, respectively. In the random forest model, 109 species achieved the mean decrease accuracy (MDA) ≥ 1.0. Finally, 19 species passed the three statistical tests and were defined as important differential species. e, Examples of important differential species. The *P* values of univariate analysis (Wilcoxon Mann–Whitney test) were labelled.

To quantify the microbial taxa and gene functions, we performed a shotgun metagenomic sequencing on a total of 68 faecal samples from 34 patients, including 25 MPRs and 9 non‐MPRs. The phylum‐level community composition in each sample is shown in Figure S3. The baseline alpha diversity showed a significantly higher level in MPR (Figure 1A) and pCR (Figure S4A) patients. PCoA and PERMANOVA test based on Aitchison distance showed a small but significant separation of the two MPR groups at baseline (Figure 1B). After treatment, these differences were all diminished. Moreover, no distinct separation was observed between the pCR groups (Figure S4B). To identify differential species that associated with the MPR, three statistical approaches were used (Method). First, we conducted a reduced multivariate linear model to adjust the imbalanced factors of age and tumour histology. Taxonomically, several *Ruminococcaceae*, *Lachnospiraceae*, and *Clostridiales* species were enriched in the MPR group, while *Prevotella* species were depleted. When further adjusting the other demographic factors, the associations still remained (Figure 1C). Taking the robustness of analysis into consideration, the univariate test and random forest modelling were further performed. Among the differential species identified by each method, 19 species demonstrated a consistent significance and were defined as important differential species (Figure 1D; Table S3). Fifteen of them have been further confirmed by the ALDEx2 method (with *p* < .05 and |Effect size| >.5, Table S4). In particular, the species belonging to the *Clostridiales* order, like *Clostridium sp. M62/1*, *Angelakisella massiliensis*, *Eisenbergiella tayi*, *Ruminococcus callidus*, *Ruminococcus bicirculans*, and *Ruminococcaceae bacterium D16* were significantly increased in the MPR group at baseline (Figure 1E). Among them, a higher abundance of *R. callidus* was also associated with a significantly longer DFS (Figure S5). Similar to the relative studies in advanced NSCLC,^28^, ^29^ *Ruminococcaceae* bacteria were positively correlated with the efficacy, while the *Lachnospiraceae* and *Clostridiales* species are rarely reported in NSCLC. Meanwhile, in contrast to our findings, the *Prevotella* bacteria are found to be enriched in responders to anti‐PD‐1 therapy in advanced malignancies.^8^

**FIGURE 2:**
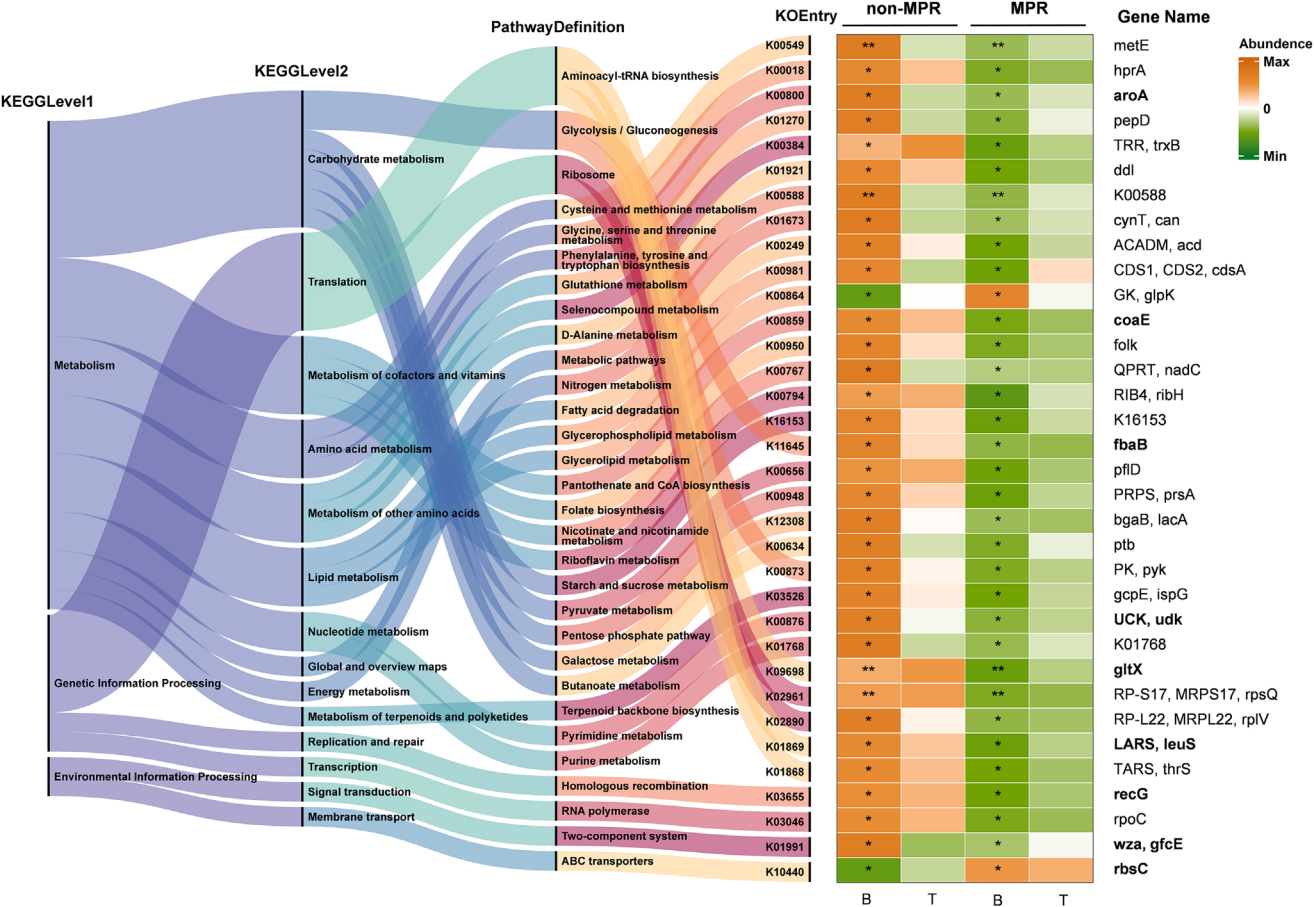
Metagenomic KO function of metabolism is associated with the major pathologic response of neoadjuvant anti‐PD‐1 immunochemotherapy. The standardized abundance of KO functions and their corresponding pathway names were visualized by a heatmap and a Sankey plot, respectively. Important differential functions investigated by univariate analysis, multivariate linear analysis and random forest were labelled in bold font. The *p*‐values of univariate analysis (Wilcoxon Mann–Whitney test) were labelled as symbols: ***p < .001; **p < .01; *p < .05.

To characterize the microbial functional profiling, the KEGG orthology (KO) genes were annotated. Unlike the major shifts in the taxonomic structure and composition, only a modest overall alteration was observed in microbial KO functions (Figure S6A). A total of 34 functions were differentially abundant between the MPR group and the non‐MPR group at baseline (Figure 2). Among them, most of the microbial genes contributing to the metabolic pathways are enriched in the non‐MPR group. Using three statistical methods to investigate the important differential functions as described above, nine KEGG orthologues remained with robust significance (Figure 2, bold font; Table S5). Specifically, the altered metabolic KO functions of the microbiome in non‐MPR patients were dominated by pathways related to the metabolism of carbohydrates, amino acids, and cofactors. These functions were mainly contributed by *Proteus mirabilis* and *Prevotella* species and negatively correlated with the butyrate‐producers such as *Coprococcus eutactus* and *Lawsonibacter asaccharolyticus* (Figure S6B). Additionally, these altered metabolic KO functions were also positively correlated with the non ‐ MPR‐related virulence factors of exoenzyme (the Mu toxin, Zn^2^⁺ metalloprotease and TlyC protein; Figure S6C). This suggests that related microbial metabolic pathways may facilitate the production or activity of these pathogenic exoenzymes, thereby impacting the therapeutic outcomes of neoadjuvant anti‐PD‐1 immunochemotherapy.

### 4.3 Plasma and faecal metabolomic signatures are associated with the major pathologic response of neoadjuvant anti‐PD‐1 immunochemotherapy

**FIGURE 3:**
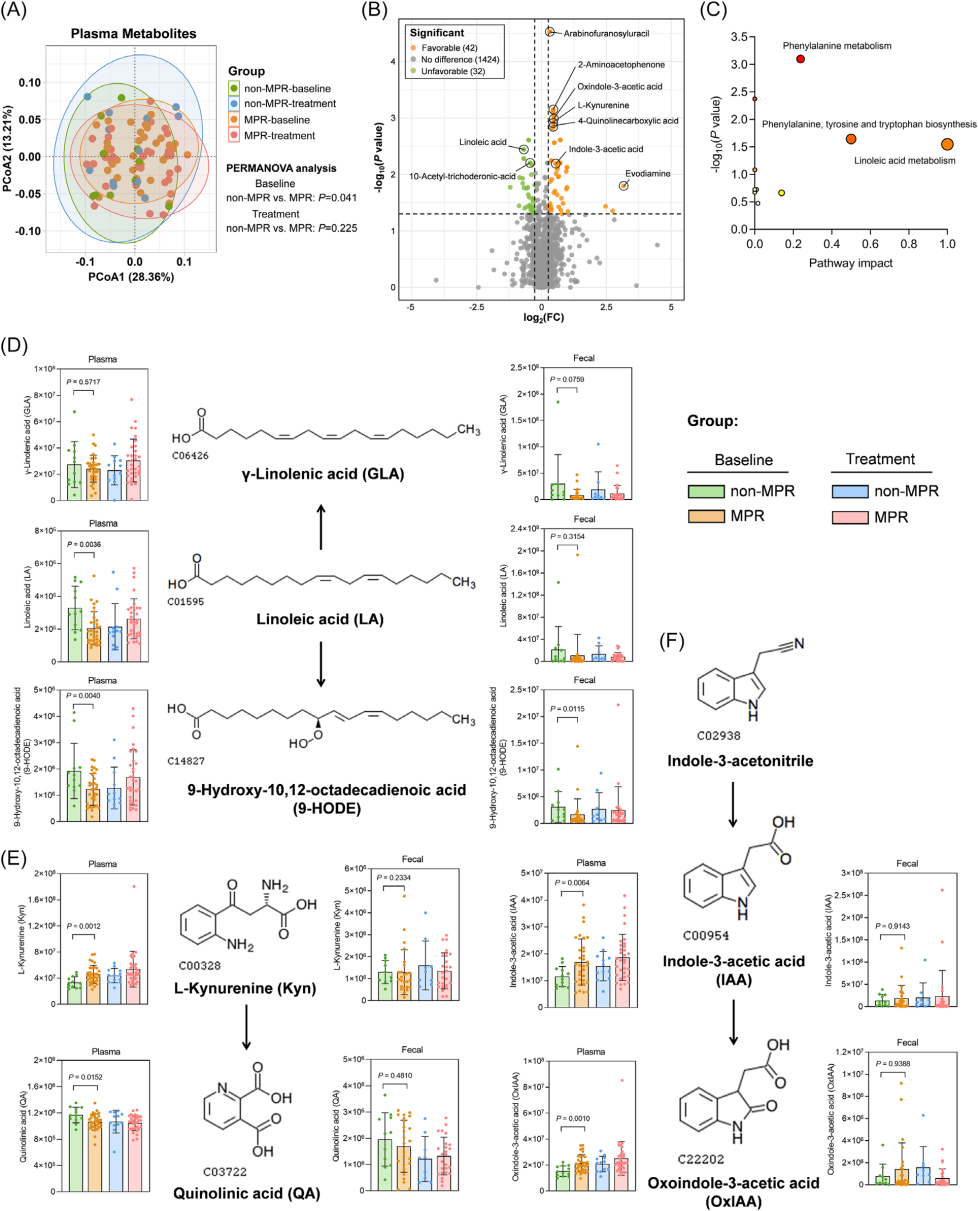
Alterations of plasma metabolomic composition and pathway in patients achieve major pathologic response. (A) PCoA plot of 1498 plasma metabolites based on Bray–Curtis distance. PERMANOVA was used for distance‐based hypothesis testing. (B) Volcano plot showing the differential metabolites related to the pathological response. The log‐transformed fold‐change (FC) was set as the x‐axis. The log‐transformed *p*‐values of univariate analysis, in which Welch's *T* test was used for normally distributed data and the Wilcoxon Mann‐Whitney test was used for non‐normal data, were set as the y‐axis. (C) KEGG pathway analysis of differential metabolites with univariate *p *< .05. The pathway impact was calculated by adding up the importance measures of each of the matched metabolites and then dividing by the sum of the importance measures of all metabolites in each pathway. Both the size and the x‐coordinate of circles indicate the pathway impact. (D–F) Plasma and faecal abundance of differential metabolites on linoleic acid and tryptophan metabolic pathways. The floating bar shows the minimum and maximum of abundance, with a line at the mean level.

To define the metabolomic profile and its relationship with the pathologic response, we surveyed the plasma and faecal metabolome using an untargeted LC‐MS method. The overall plasma metabolomic profile showed slight differences between the MPR and non‐MPR patients at baseline (Figure 3A), while the faecal metabolome showed no significant differences (Figure S7A). Similarly, no distinct separation was observed between the pCR groups (Figure S8A,B). Based on the univariate test, a total of 74 plasma metabolites were found to be associated with the major pathologic response of patients (Figure 3B). The differential metabolites with univariate *p* < .05 were mainly enriched on the metabolomic pathways of linoleic acid and aromatic amino acid, including tryptophan, tyrosine and phenylamine (Figure 3C). It is different from the previous metabolomic studies on advanced NSCLC, which have shown that the hypoxanthine, lactate, histidine and alanine metabolites are predictive biomarkers of responses to anti‐tumour immune therapy.^10^, ^11^, ^12^ When using the multivariate linear model and random forest model to further control the related covariates (Method), 19 differential features, including linoleic acid (LA) and 9‐hydroxyoctadeca‐10,12‐dienoic acid (9‐HODE), retained significant associations (Table S6; Figure S7B).

Particularly, the plasma LA showed significantly increased levels in non‐MPR patients at baseline, and its metabolite γ‐linolenic acid (GLA) in faeces and 9‐HODE in both plasma and faeces were also significantly enriched in the non‐MPR group (Figure 3D). In the aromatic amino acid metabolism, L‐kynurenine (Kyn) showed an increased baseline abundance in the plasma of MPR patients, while quinolinic acid (QA) was found to be an unfavourable metabolite that was depleted in the MPR group (Figure 3E). The indole‐3‐acetic acid (IAA) and its oxidative metabolite oxindole‐3‐acetic acid (OxIAA) were significantly enriched in the plasma of MPR patients at baseline. This favourable relationship could also be observed in the faecal metabolome (Figure 3F). Moreover, higher baseline levels of IAA in plasma were also associated with the complete pathologic response (Figure S8C) and a longer DFS (Figure S9).

### 4.4 Interplays between differential metabolites and gut microbiome suggest functional links on the neoadjuvant anti‐PD‐1 immunochemotherapy

**FIGURE 4:**
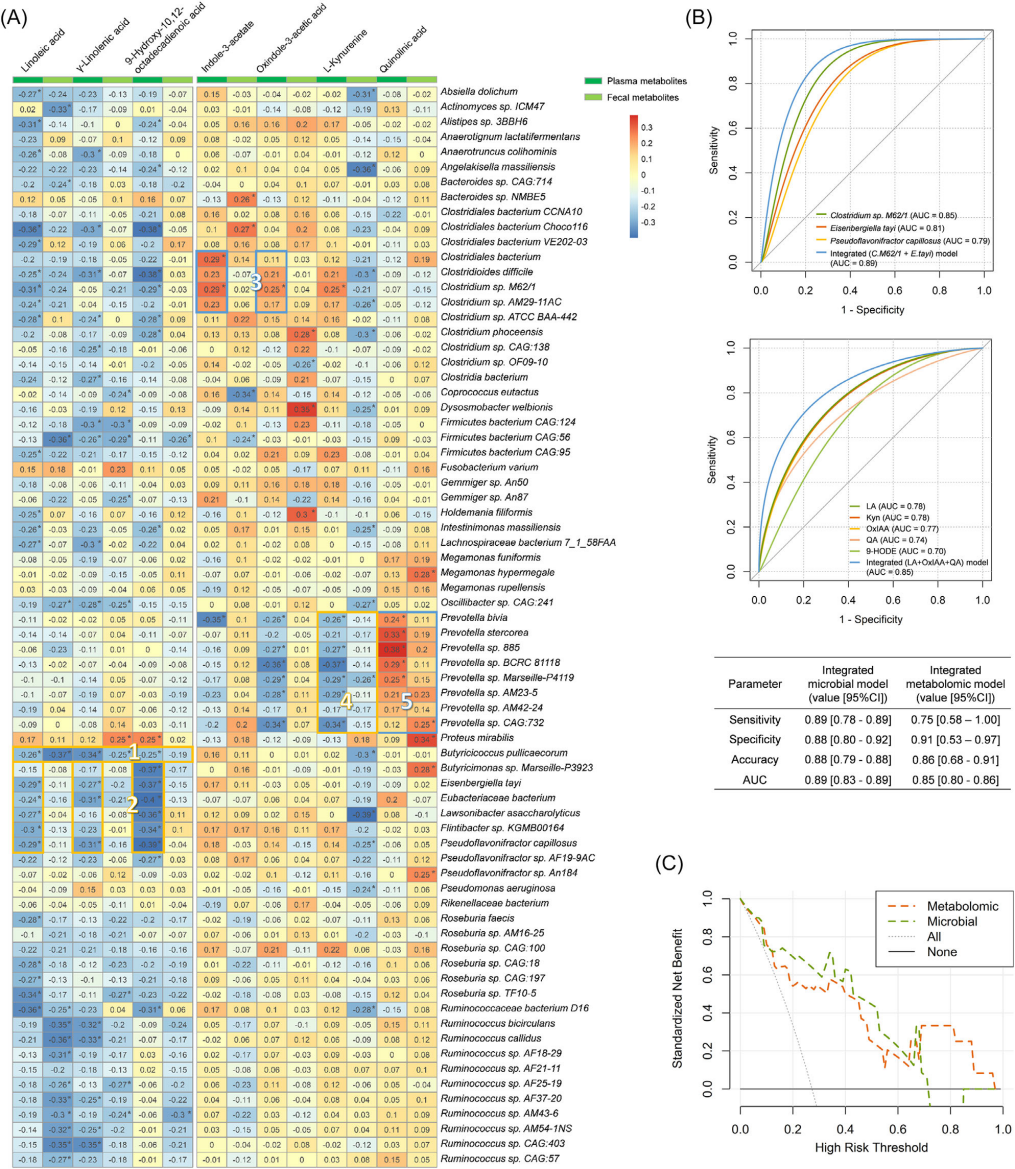
Correlations between differential metabolites and gut microbiome and the predictive performance of these features. (A) Heatmap of the Spearman's rank correlation of differential species and metabolites that conducted using the pairwise data from all sampling times. The coefficient *R* was shown on the plot. *p* < .05 was labelled as the symbol *. Red squares indicate positive associations, while blue squares indicate negative associations. (B) Receiver operating characteristic (ROC) curve of logistic regression model based on the selected differential species/metabolite, or their integration. The integrated microbial model was developed in the baseline microbial dataset (*n *= 34) and consists of *Clostridium sp. M62/1* (*C. M62/1*) and *Eisenbergiella tayi* (*E. tayi*). The integrated metabolomic model was developed in the baseline metabolomic dataset (*n *= 44) and includes linoleic acid (LA), oxindole‐3‐acetic acid (OxIAA) and quinolinic acid (QA). (C) Decision curve analysis for the integrated microbial model (green dashed line) and the integrated metabolomic model (orange dashed line). The grey dotted line represents the assumption that no patient achieves MPR. The black solid line represents the assumption that all patients achieve MPR.

We functionally characterized the differential species and metabolites by investigating their relationships across the whole samples (Tables S8 and S9). A variety of microbe‐metabolite correlation clusters were detected (Figure 4A). For example, linoleic acid and its metabolites were negatively correlated with the butyrate‐producer *Butyricicoccus pullicaecorum* (cluster 1). Plasma levels of these metabolites were also inversely related to several other butyrate‐producing species, including *Eubacteriaceae bacterium*, *Lawsonibacter asaccharolyticus*, and *Pseudoflavonifractor capillosus* (cluster 2). These relationships also exhibit significant distance dependencies tested by the dCor method (Figure S10). Consistent with the role of *Clostridia* species in producing indolic compounds like IAA,^30^ the plasma IAA and OxIAA were found to be positively correlated with several *Clostridium* species, like *C. M62/1* (cluster 3), and is distance‐correlated with *Clostridium sp. AM29‐11AC* (Figure S10). In addition, a cluster of negative associations was observed between *Prevotella* species and the Kyn (cluster 4), whereas this group of microbes was positively correlated with QA (cluster 5).

By using logistic regression, we finally evaluated the predictive potential of differential species or metabolites for the major pathologic response of NSCLC neoadjuvant anti‐PD‐1 immunochemotherapy (Figure 4B; Table S10). All of the labelled features could make accurate predictions with an AUC above .70. The integrated model consisted of *Clostridium sp. M62/1* and *E. tayi* (integrated microbial model), or LA, OxIAA, and QA (integrated metabolomic model) showed optimal predictions with AUC values of .89 [95%CI, .83– .89] and .85 [95%CI, .80– .86], respectively. The calibration curve and HL goodness‐of‐fit test also indicated no departure from perfect fit for both models (*p* = .86 and .29, respectively; Figure S11). In 10‐fold cross‐validation, the models distinguished the MPR from non ‐ MPR patients with accuracies of .82 [95%CI, .76– .85] and .77 [95%CI, .74– .81], respectively. To determine the clinical usefulness of the two models, a decision curve analysis was conducted. The integrated microbial model could provide higher net benefits than the baseline strategy at threshold probabilities of .1 and .7, while the integrated metabolomic model could obtain a broader clinical benefit when the threshold probabilities are above .1 (Figure 4C). Moreover, a Cox regression model consisting of LA, OxIAA and QA could also well‐predict the 1‐year and 2‐year DFS, achieving AUCs of .79 [95%CI, .69– .90] and .74 [95%CI, .57– .92], respectively (Figure S12).

## 5 DISCUSSION

Neoadjuvant anti‐PD‐1 immunochemotherapy has been increasingly incorporated into the perioperative treatment of resectable NSCLC, but it has not consistently yielded durable responses. In the current study, we found that the gut microbial diversity and composition, as well as the plasma metabolomic profiles at baseline, could distinguish the major pathological response of combined anti‐PD‐1 therapy. Specifically, the species belonging to the *Clostridiales*, *Lachnospiraceae* and *Ruminococcaceae* family such as *C. M62/1*, *A. massiliensis*, *E. tayi*, *R. callidus*, *R. bicirculans*, and *R. bacterium D16* are significantly enriched in the MPR group. Among them, the *C*. *M62/1* is found to be positively correlated with the plasma IAA and OxIAA, which are also defined as favourable metabolites. Conversely, butyrate‐producers such as *E. tayi* and *B. pullicaecorum* are negatively correlated with the plasma LA metabolites. Collectively, these results emphasize the important roles of gut microbiome composition and metabolic homeostasis in the neoadjuvant anti‐PD‐1 immunochemotherapy.

To our knowledge, this is the first study investigating the signatures of gut microbiome and metabolome in the neoadjuvant anti‐PD‐1 immunochemotherapy of early‐stage NSCLC. Previously, many intestinal microbiome studies have been conducted in advanced NSCLC. In 2018, Routy et al.^28^ reported that commensals such as *Ruminococcus*, *Alistipes*, *Eubacterium*, and *Akkermansia* bacteria are enriched in the responders receiving ICI treatment. The following year, Jin et al.^8^ studied the gut microbial diversity in Chinese NSCLC patients and found that *Alistipes putredinis*, *Bifidobacterium longum*, and *Prevotella copri* were positively correlated with the effectiveness of anti‐PD‐1 therapy. In 2022, Derosa et al.^7^ further confirmed that *Akkermansia muciniphila*, accompanied by *B. adolescentis*, could predict the PD‐1 blockade efficacy in advanced NSCLC. In our study, we have also performed analysis focusing on these well‐established microbial biomarkers, including *A. muciniphila*, *B. adolescentis*, and *B. longum*, and found a consistent but not significant enrichment trend in MPR patients (Figure S13A). Incorporating these prior metagenomic classification tools did not improve our model performance in predicting the MPR (Figure S13B), but the consistent trends of these species supported the biological plausibility of our cohort, which exhibits no counterintuitive biases. However, some previous studies in advanced malignancy identified the pro‐inflammatory *Prevotella* as the favourable species in anti‐PD‐1 treatment,^8^, ^31^ while our investigation showed that they are unfavourable. This discrepancy may be attributed to the inherent differences in disease stage and reflects the stage‐specific biology. Pro‐inflammatory cytokines are thought to be the promoters of early tumorigenesis, which could lead to immunosuppression.^32^ Therefore, the baseline enrichment of pro‐inflammatory features was linked to the worse prognosis in our early‐stage NSCLC patients. However, as Marcelo's team proposed, although the baseline inflammation causes immunosuppression, triggering pro‐inflammatory response following the initiation of anti‐PD‐1 treatment may enhance the therapeutic efficacy by expanding the higher numbers of CD8+ T cells.^33^ Since advanced/metastatic malignancies usually have decreased inflammatory features,^34^ the pro‐inflammatory microenvironment (associated with increased *Prevotella*) may be favourable for the therapy of advanced/metastatic disease. Beyond the above reasons, the different cohort characteristics (e.g., geographic, genetic or dietary factors) or methodological factors (e.g., sampling points or sample sizes) may also contribute to this inconsistency, which needs to be assessed in a further comparative study. Nevertheless, the opposing trend of pro‐inflammatory species found in advanced NSCLC and the current early‐stage NSCLC provides an insight that the future development of gut microbial biomarkers or therapeutics may be tailored according to the disease staging.

In addition to the intestinal microbial biomarkers, our study also highlighted several important differential metabolites that were significantly associated with the therapeutic outcome of neoadjuvant anti‐PD‐1 immunochemotherapy. The differential metabolites we identified were also mainly immune‐related. Accumulating evidence has found that the n‐6 long‐chain polyunsaturated fatty acids (LC‐PUFAs), including the LA and its metabolites that are significantly enriched in non‐MPR patients, are primarily pro‐inflammatory and tumorigenic.^35^ LA is the essential fatty acid that is mainly obtained from food (and can be co‐metabolized by the host and microbes), which is then metabolized to GLA and then on to arachidonic acid to promote inflammation.^36^ Though not significant, an increased abundance of arachidonic acid could be observed in the non‐MPR group as well (Table S6). Additionally, the oxidative derivatives of LA, like 9‐HODE and 13‐HODE, can also impact the inflammation and tumour proliferation depend on their enantiomeric forms.^37^

Tryptophan metabolism is also closely related to tumour immunity. Metabolites generated from tryptophan, such as Kyn, QA and IAA, are potent ligands of the aryl hydrocarbon receptor (AhR) that are involved in the regulation of the immune system.^38^, ^39^, ^40^ Among them, IAA is primarily produced by plants (which can be obtained through diet) or by intestinal bacteria such as *Clostridium bartlettii* and *C*. *M62/1*,^41^ as supported by our correlation analysis. The neutrophil‐derived oxidation of IAA is reported to induce the accumulation of ROS and the downregulation of autophagy that compromise the proliferation of cancer cells,^42^ which may explain the identified enrichment of IAA and OxIAA in MPR patients. It should be noted that the generation of OxIAA is currently regarded to be primarily carried out by plants, with some contribution from certain bacteria.^43^, ^44^ It is unclear whether the IAA oxidation in neutrophils can produce the OxIAA and whether it can play a role in anti‐tumour immunity. In contrast to IAA, QA is found to have deleterious effects on the host immune response. Tumour cells accumulated QA to prevent ROS‐induced apoptosis (by synthesizing NAD(+)) and prime macrophage polarization, which collectively promote the tumour immune tolerance.^45^, ^46^

Based on the hypothesis that gut microbiota‐derived metabolites spread from their original location and impact the systemic levels of metabolites, we combined gut microbiome analysis with faecal metabolomics and plasma metabolomics in an NSCLC anti‐PD‐1 clinical study. However, the results showed that both the correlations between gut microbiota and faecal metabolites and the associations between responses and faecal metabolites were generally weaker than those with plasma metabolites. On one hand, the component of faeces is mainly determined by food intake, rather than microbial metabolism. On the other hand, nutrients and gut microbiota‐derived metabolites, especially the lipid, bile salt, vitamin and some amino acids, are mainly absorbed in the small intestine.^47^ Following absorption, the faecal levels of metabolites may be varied from the original levels produced by microbiota. Accordingly, when studying the gut microbial metabolism, appropriate sampling sites may be important.

In our study, only baseline microbial profiles were associated with MPR. The most plausible explanation is that the neoadjuvant immunochemotherapy caused a perturbation to the gut microbiomes in all patients, potentially driving them towards a similar condition regardless of the initial baseline differences. Additionally, the treatment‐induced dysbiosis may further increase the variability within each group, making the differences harder to detect with our sample size.

Several limitations should be noted for the study. As of March 2025, this study has not yet reached the median DFS. Therefore, the findings on both the biomarker‐DFS associations and the metabolomic Cox regression model should be interpreted cautiously. A longer follow‐up is necessary to fully evaluate the predictive value of the identified biomarkers. In addition, the current study was conducted in small cohorts, which may result in potential bias. A larger and independent cohort is needed to rigorously validate the differential features and their predictive performance. Meanwhile, dietary factors need to be collected and controlled in the validation study. In addition, the functional mechanisms underlying the microbe‐metabolite interactions were currently speculated based on associative analysis. Direct experimental verification is needed to confirm whether they have causal relationships and how they work. Future studies that quantify these differential features and link them with immune cell infiltration profiles or cytokine levels may help validate their roles in the anti‐tumour immune response.

In conclusion, our metagenomic and metabolomic study found the conspicuous associations between baseline microbiome/metabolome composition and the pathologic response in early‐stage NSCLC patients, which suggested a potential interventional strategy in improving the efficiency of neoadjuvant anti‐PD‐1 immunochemotherapy. Furthermore, correlations of differential species and metabolites we identified also provided new insights into the underlying mechanisms. A small panel of gut microbiota (*Clostridium* sp. *M62/1* and *E. tayi*) or plasma metabolites (LA, OxIAA, and QA) could achieve the promising prediction of the pathologic response. Additional research on the mechanism verification and large‐scale cohort validation holds the potential benefit of probiotic manipulation or targeted metabolism interventions in NSCLC neoadjuvant anti‐PD‐1 immunochemotherapy.

## AUTHOR CONTRIBUTIONS

Hao Long, Xueding Wang, and Yuheng Zhou designed the research. Hao Long and Yaobin Lin recruited the patients. Ailing Cao, Yanping Guan, and Yiyu Zhang performed experiments. Wenyu Zhai, Yuheng Zhou, and Shoucheng Feng collected clinical data. Ailing Cao and Shaoxing Guan analyzed results and made the figures. Ailing Cao and Yaobin Lin wrote the first draft of the manuscript. Xueding Wang and Min Huang provided valuable advice in revising the manuscript. All authors reviewed the manuscript and approved the final version.

## CONFLICT OF INTEREST STATEMENT

The authors declare no conflict of interest.

## CONSENT FOR PUBLICATION

Written informed consent was directly obtained from all patients.

## ETHICS STATEMENT

This trial was approved by the Ethics Committee and Institutional Review Board of Sun Yat‐sen University Cancer Center (A2020‐008). The procedures used in this study adhere to the tenets of the Declaration of Helsinki.

## Supporting information



Supporting Information

Supporting Information

Supporting Information

## Data Availability

The datasets generated and analyzed during the current metagenomic study are available in the NCBI SRA repository, PRJNA1226730. Other datasets used and analyzed during the current study are available from the corresponding author on reasonable request.
