# Supplementary material for: Baseline multi‐omics signatures could predict therapeutic response to neoadjuvant anti‐PD‐1 immunochemotherapy in non‐small‐cell lung cancer
Source: Clin Transl Med. 2026 Jan 7;16(1):e70579. doi: 10.1002/ctm2.70579 (PMC12778419; doi:10.1002/ctm2.70579)
Supplement: Supplementary file 1 — Supporting Information [file CTM2-16-e70579-s002.pdf]

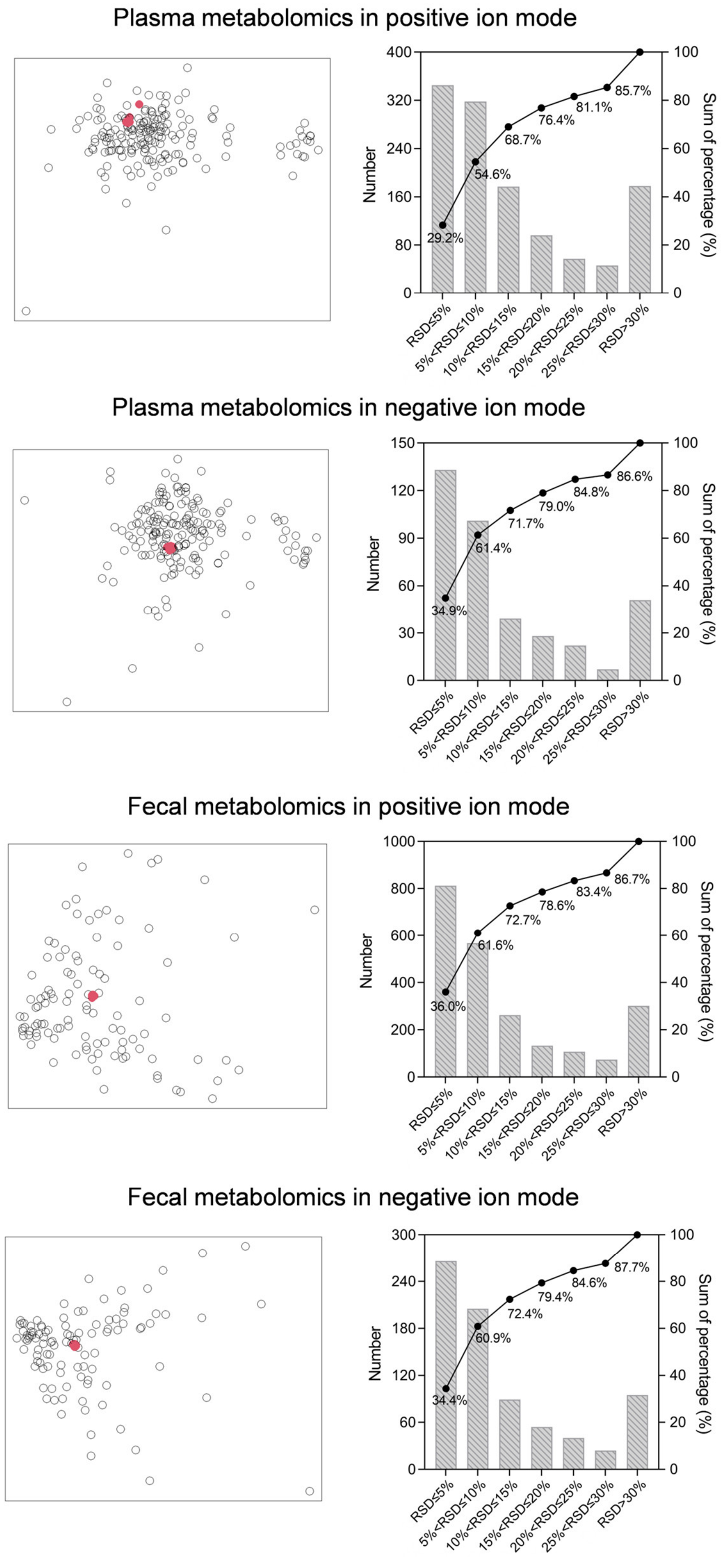

Fig.S1 Principal component analysis score plots (left) and relative standard deviation of QC samples (right) that illustrating the quality of metabolomic detection

In the principal component analysis plots, QC samples are represented as red dots, while other samples are black dots.

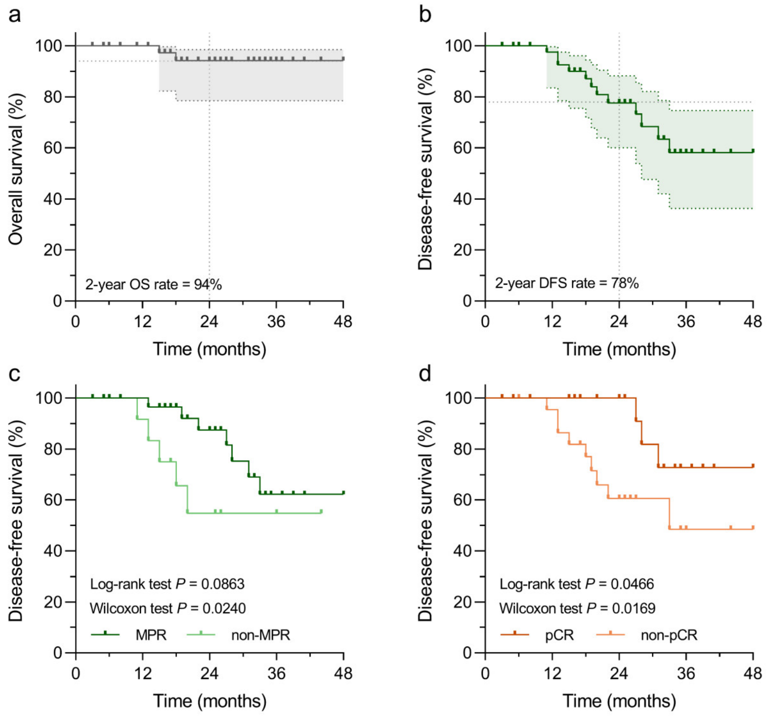

Fig.S2 Overall survival (OS) and disease-free survival (DFS) among all patients and patients with different pathological responses.

a, and b, With the median follow-up of 24.5 months, the 2-year OS and DFS rates were 94% and 78%, respectively.

c, The DFS among patients with major pathologic response (MPR) or not (non-MPR).

d, The DFS among patients with pathologic complete response (pCR) or not (non-pCR).

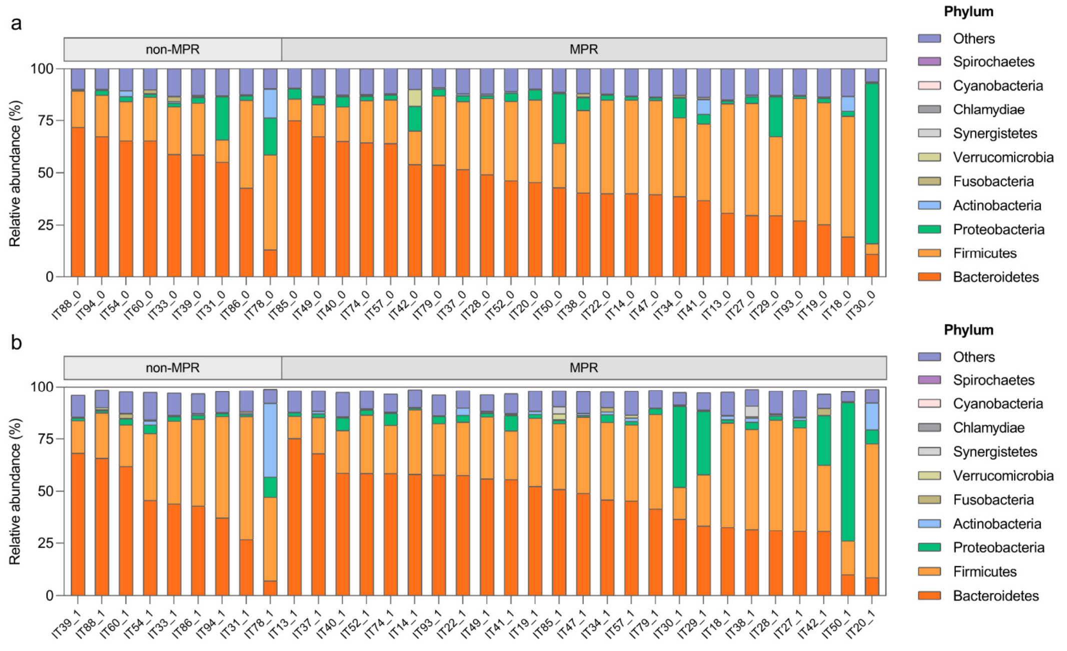

Fig.S3 The microbial community composition at the phylum level.

a, The relative abundance of microbial community at the phylum level in baseline samples.

b, The relative abundance of microbial community at the phylum level in samples collected before surgery.

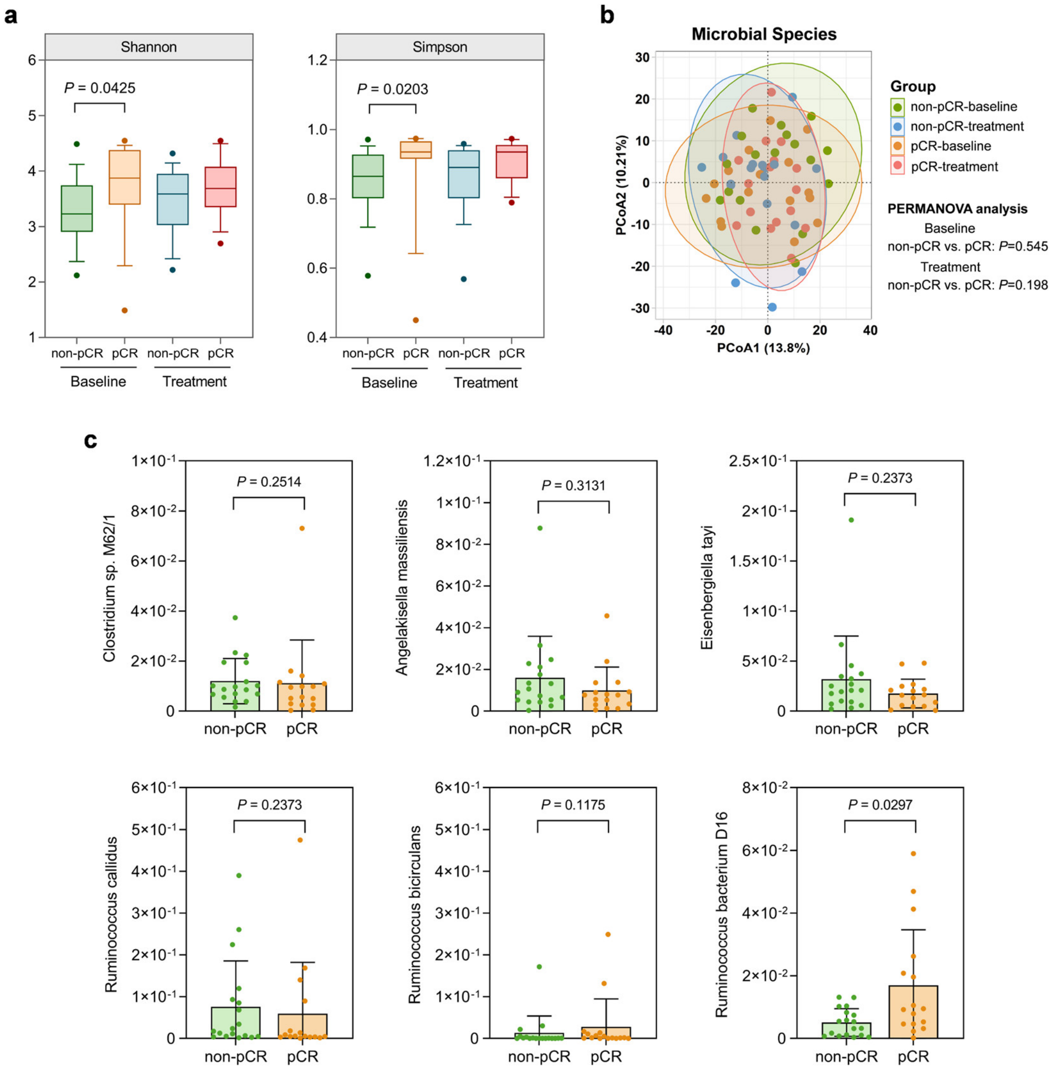

Fig.S4 Association of microbiota signatures with the complete pathologic response

a, The indices of alpha-diversity in patients achieved complete pathologic response (pCR) or not (non-pCR). Shannon and Simpson values were compared using Wilcoxon Mann-Whitney test.

b, The beta-diversity showing the overall compositional differences between groups. Principal coordinate analysis (PCoA) was based on Aitchison distance and tested by permutational multivariate analysis of variance (PERMANOVA).

c, The relationships between the baseline abundance of important differential species identified in MPR analysis and the complete pathologic response.

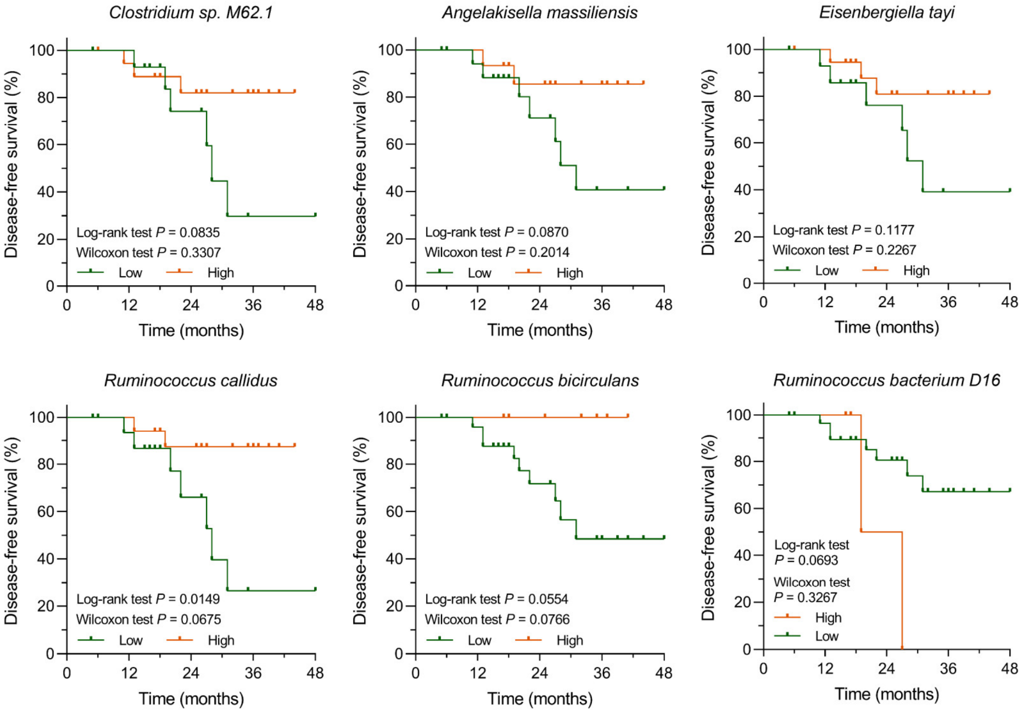

Fig.S5 Association between important differential species and the disease-free survival

The Kaplan-Meier survival plots were stratified by the optimal cut-off of species abundance at baseline.

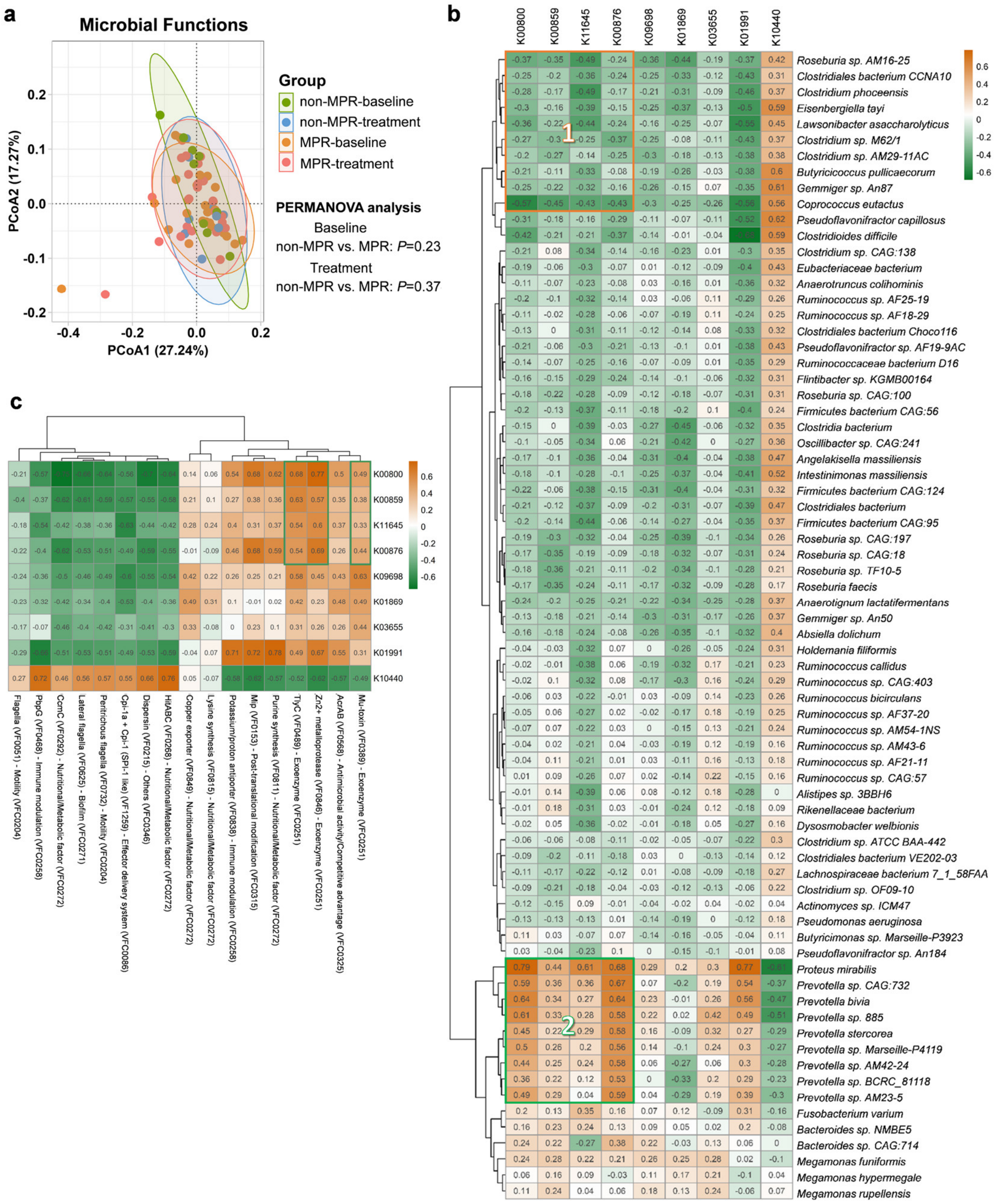

Fig.S6 Alteration of microbial functional profiles and its association with both taxonomic composition and virulence factors (VFs)

a, PCoA based on Aitchison distance showed a moderate overall microbial functional difference at baseline.

b, Heatmap of the Spearman's rank correlation of species and differential KO functions. The coefficient *R* was shown on the plot. Orange squares indicate positive associations while green squares indicate negative associations.

c, Heatmap of the Spearman's rank correlation of VFs and differential KO functions. VFs were annotated by aligning the metagenomic dataset against the Virulence Factor Database (VFDB). The plotted VFs were all significantly associated with the major pathologic response (Wilcoxon Mann-Whitney test $P < 0.05$). The coefficient *R* was shown on the plot. Orange squares indicate positive associations while green squares indicate negative associations.

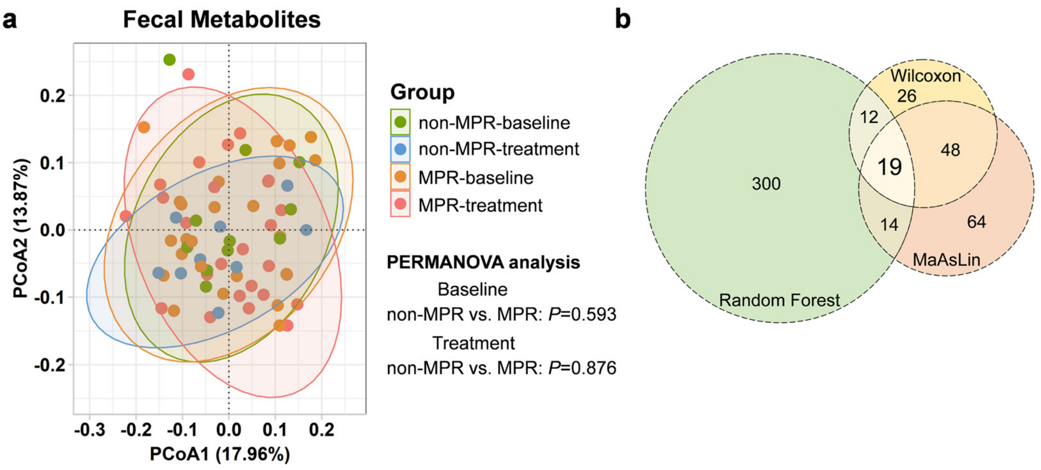

Fig.S7 Fecal and plasma metabolomic signatures associated with the major pathologic response

a, PCoA plot of 2745 fecal metabolites based on Bray-Curtis distance. PERMANOVA was used for distance-based hypothesis testing.

b, The number of differential plasma metabolites tested by three statistical methods. A total of 105 and 145 metabolites achieved a *P* value of < 0.05 in univariate analysis and multivariate linear analysis, respectively. In random forest model, 345 metabolites achieved the mean decrease accuracy (MDA) ≥ 1.0. Finally, 19 metabolites passed the three statistical test and were defined as important differential species.

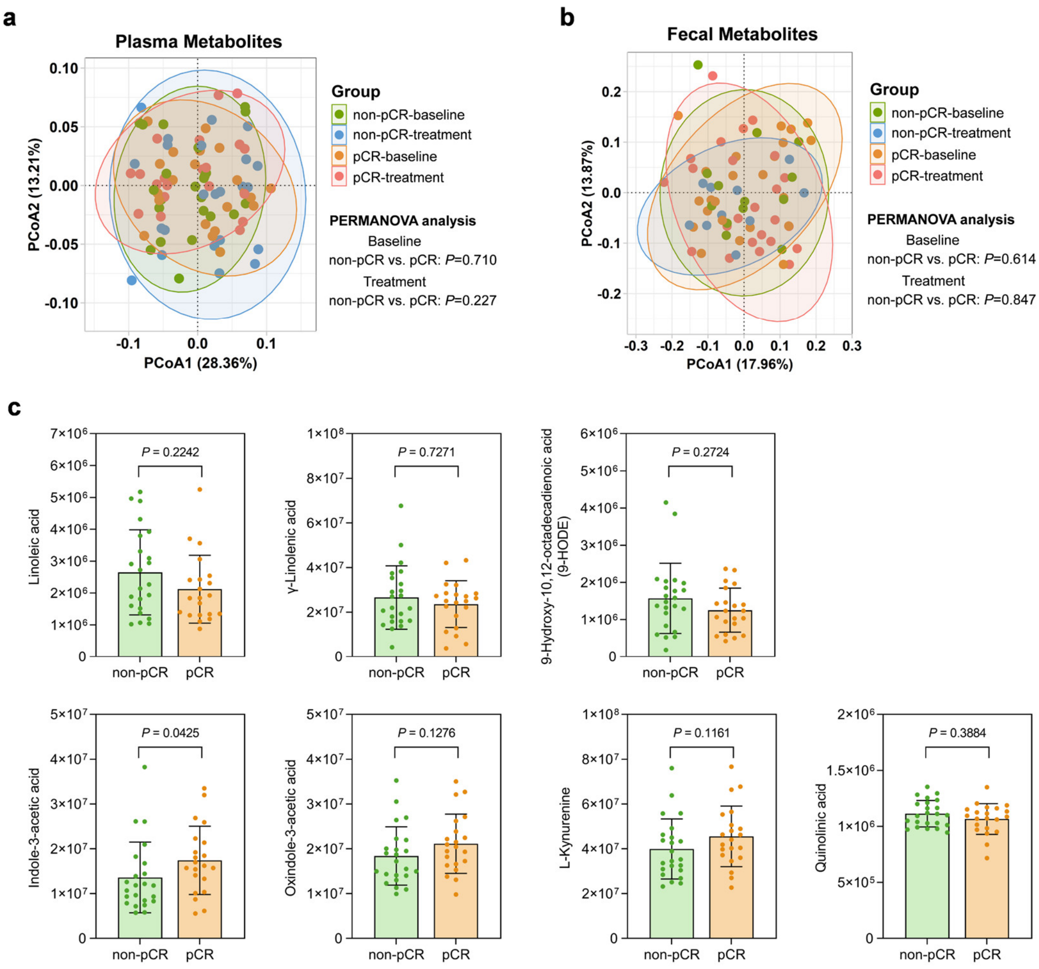

Fig.S8 Association of metabolomic signatures with the complete pathologic response

a, Principal coordinate analysis (PCoA) plot of plasma metabolites showing the overall compositional differences between patients achieved complete pathologic response (pCR) or not (non-pCR). PERMANOVA was used for distance-based hypothesis testing based on Bray-Curtis distance.

b, PCoA plot of fecal metabolites showing the overall compositional differences between pCR and non-pCR patients. PERMANOVA was used for distance-based hypothesis testing based on Bray-Curtis distance.

c, The relationships between the baseline levels of differential metabolites identified in MPR analysis and the complete pathologic response.

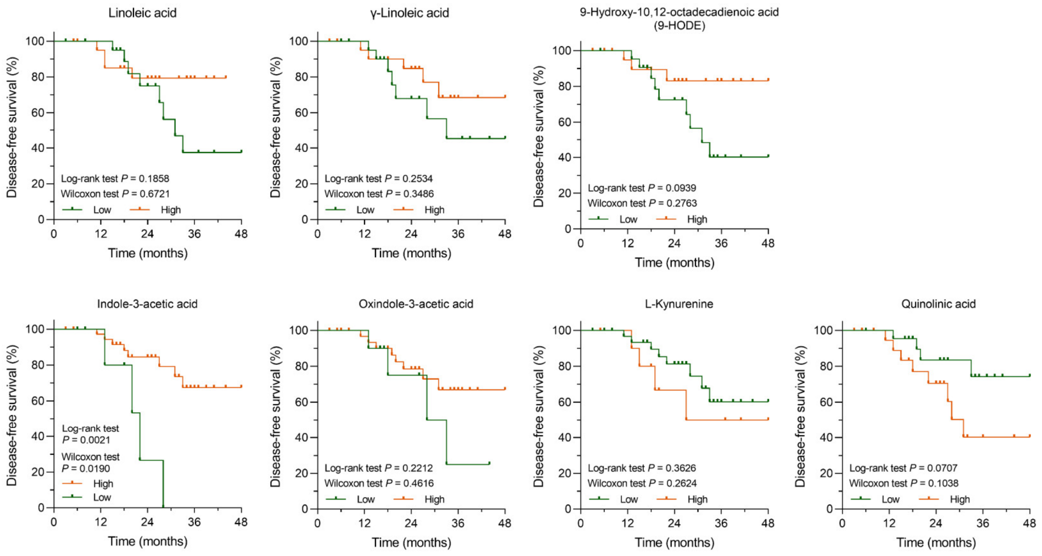

Fig.S9 Association between differential plasma metabolites and the disease-free survival

The Kaplan-Meier survival plots were stratified by the optimal cut-off levels of plasma metabolites at baseline.

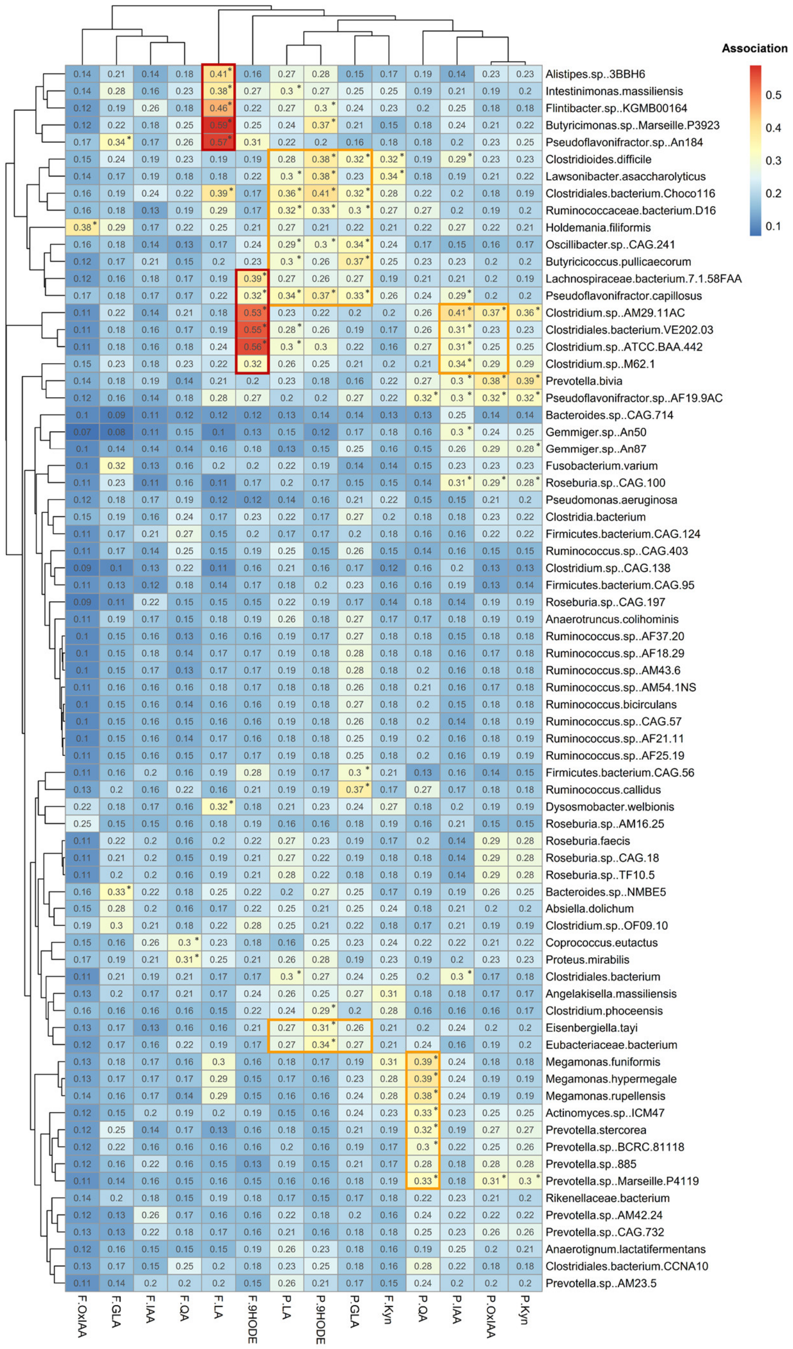

Fig.S10 Distance correlations between differential metabolites and gut microbiome

Heatmap of the distance correlation of differential species and metabolites. The association coefficient was shown on the plot. *P* < 0.05 was labeled as the symbol *.

Abbreviations: F.OxIAA, Fecal oxindole-3-acetic acid; F.GLA, Fecal γ-linolenic acid; F.IAA, Fecal indole-3-acetic acid; F.QA, Fecal quinolinic acid; F.LA, Fecal linoleic acid; F.9HODE, Fecal 9-hydroxyoctadeca-10,12-dienoic acid; P.LA, Plasma linoleic acid; P.9HODE, Plasma 9-hydroxyoctadeca-10,12-dienoic acid; P.GLA, Plasma γ-linolenic acid; F.Kyn, Fecal L-kynurenine; P.QA, Plasma quinolinic acid; P.IAA, Plasma indole-3-acetic acid; P.OxIAA, Plasma oxindole-3-acetic acid; P.Kyn, Plasma L-kynurenine.

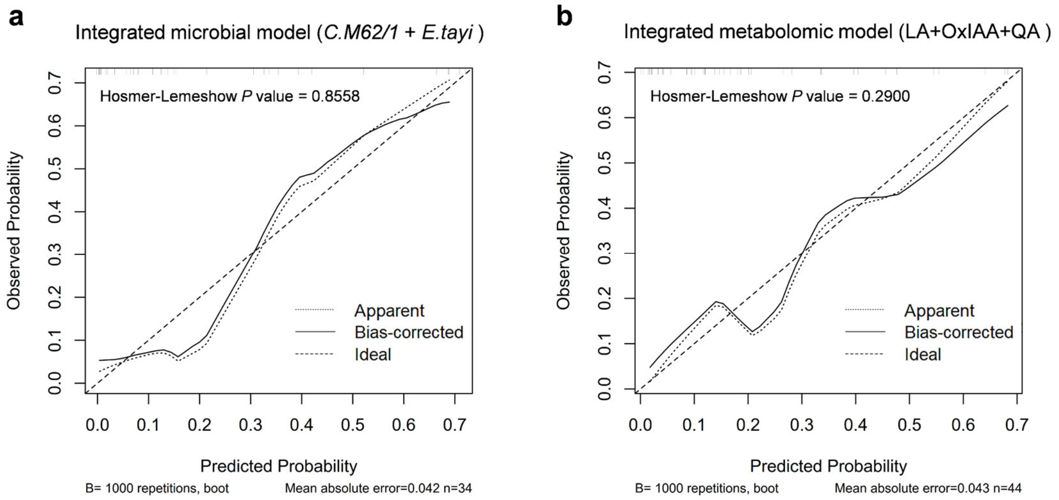

Fig.S11 The calibration curves of the integration microbial (*C.M62/1 + E.tayi*) model and integration metabolic (LA+OxIAA+QA) model for predicting the major pathologic response. The diagonal dashed line represents the perfect prediction of an ideal model. The solid line indicates the performance of the models, with a closer proximity to the diagonal dashed line signifying better predictive accuracy. The *P* values of Hosmer-Lemeshow (HL) test are labeled. A nonsignificant result (*P*>0.05) indicates no departure from a perfect fit.

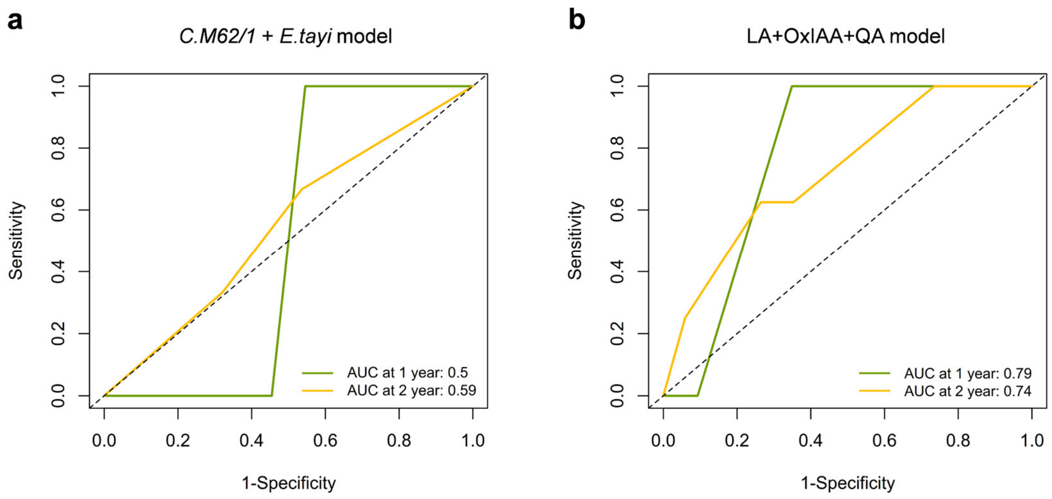

Fig.S12 Time-dependent ROC curves of the integration microbial (*C.M62/1 + E.tayi*) model and integration metabolic (LA+OxIAA+QA) model for predicting the 1-year or 2-year DFS.

The models were developed using the Cox proportional hazard regression. The prediction accuracy for the disease-free survival was assessed by the AUCs of time-dependent ROC curve.

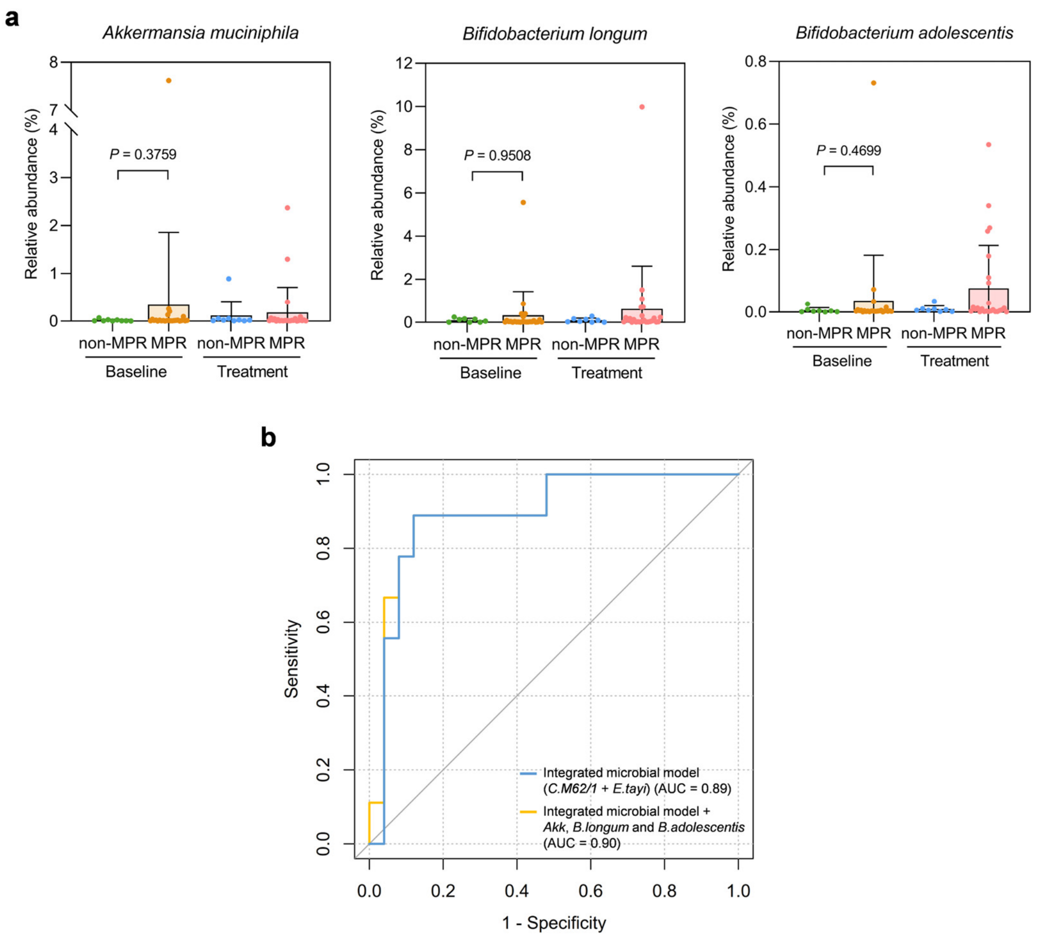

Fig.S13 Associations of *Akkermansia muciniphila*, *Bifidobacterium longum* and *Bifidobacterium adolescentis* with the major pathologic response.

a, The abundance of *Akkermansia muciniphila*, *Bifidobacterium longum* and *Bifidobacterium adolescentis* in different MPR groups. The *P* values of Wilcoxon Mann-Whitney test were labeled.

b, ROC curve of the logistic regression model incorporating *Akkermansia muciniphila* (*Akk*), *Bifidobacterium longum* (*B.longum*) and *Bifidobacterium adolescentis* (*B.adolescentis*) into the integrated microbial model.
