# Supplementary material for: Baseline multi‐omics signatures could predict therapeutic response to neoadjuvant anti‐PD‐1 immunochemotherapy in non‐small‐cell lung cancer
Source: Clin Transl Med. 2026 Jan 7;16(1):e70579. doi: 10.1002/ctm2.70579 (PMC12778419; doi:10.1002/ctm2.70579)
Supplement: Supplementary file 3 — Supporting Information [file CTM2-16-e70579-s003.docx]

This study is a subgroup analysis of LungMark research (NCT05244837), an investigator-initiated, single-arm, multicentre prospective clinical trial to explore the related biomarkers for safety and efficacy of the combination of chemotherapy and tislelizumab in NSCLC. In our study, we focused on **Cohort A** to explore the related efficacy biomarkers through metagenomic and metabolomic analyses. The full study protocol of LungMark research is listed as follow.

**Study Protocol**

| **Study Title** | Predictive Biomarker for the Efficacy and Safety of the Combination of Chemotherapy and Tislelizumab in Non Small Cell Lung Cancer：a Multicentre Prospective Clinical Trial |
| --- | --- |
| **Organization**  **in Charge** | Sun Yat-sen University Cancer Center |
| **Study**  **Endpoints** | **Primary endpoint: Drug safety incidents**  Safety as measured by number of participants with Grade 3 and 4 lab abnormalities, as defined by CTCAE v5.0.  **Secondary** **endpoint: Major pathologic response (MPR)**  The MPR is defined as less than 10% tumor cells in the pathologically resected specimen.  **Secondary** **endpoint: Pathologic complete response (pCR)**  The pCR is defined as no tumor cells observed in pathologically resected specimens.  **Secondary** **endpoint: Resectable rate**  The resectable rate is defined as the number of patients who underwent surgical resection/the number of patients in each group.  **Secondary** **endpoint: Disease-free survival (DFS)**  The DFS is defined as the interval from the surgery to the observation of confirmed disease recurrence. |
| **Study Subjects** | Initially diagnosed with stage IIB-IV NSCLC (as defined by the American Joint Committee on Cancer, 8th edition). |
| **Study Design** | 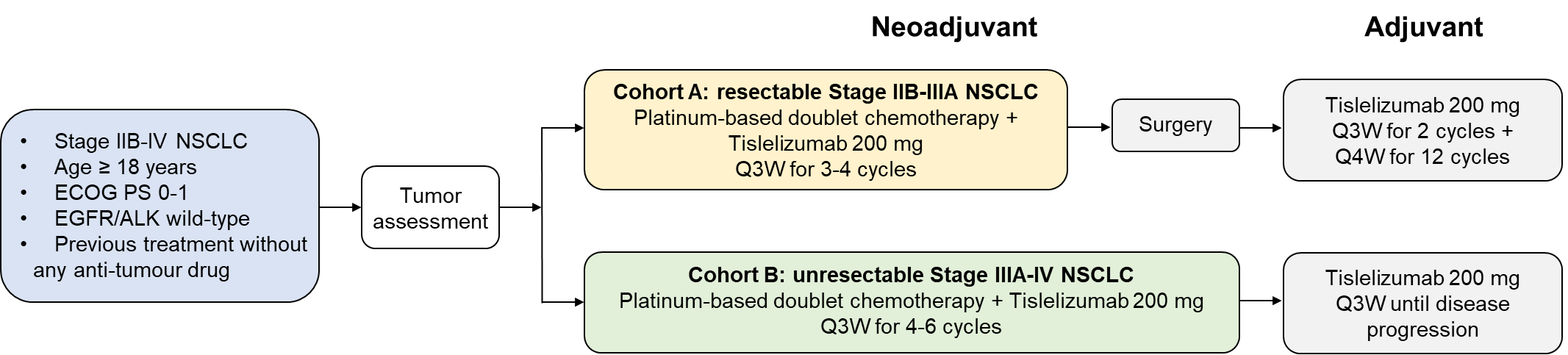 |
| **Dosing Regimen** | **Cohort A: Resectable Stage IIB-IIIA NSCLC**  Patients received neoadjuvant treatment with Platinum-based doublet chemotherapy plus Tislelizumab (200 mg) on day 1 of each 21-day cycle, for 3-4 cycles before surgical resection, followed by adjuvant intravenous Tislelizumab monotherapy for 1 year (200 mg every 3 weeks for 2 cycles, followed by 200 mg every 4 weeks for 12 cycles).  **Cohort B: Unresectable Stage IIIA/IIIB/IIIC or IV NSCLC**  Patients received neoadjuvant treatment with Platinum-based doublet chemotherapy plus Tislelizumab (200 mg) on day 1 of each 21-day cycle, for 4-6 cycles, followed by adjuvant intravenous Tislelizumab monotherapy (200 mg every 3 weeks), until disease progression or intolerable toxicity If surgery is not possible. |
| **Inclusion**  **Criteria** | 1. 1. Able to provide written informed consent and able to understand and agree to comply with study requirements and evaluation forms. 2. 2. At least 18 years of age on the date the informed consent is signed. 3. 3. At least 1 measurable lesion as defined by RECIST v1.1 criteria. 4. 4. Eastern Cooperative Oncology Group (ECOG) performance status of 0 or 1. 5. 5. Eligible for platinum-based doublet chemotherapy. 6. 6. Pre-treatment tumor tissue samples for biomarker analysis can be provided. 7. 7. Adequate hematology and end-organ function as defined by the following laboratory values (≤ 7 days prior to first dose): 8. (1) Patients did not require blood transfusion, platelet transfusion, or growth factor support ≤ 14 days prior to blood draw: i. absolute neutrophil count ≥ 1.5 × 10^9^/L, ii. Platelets ≥ 100 × 10^9^/L, iii. Hemoglobin ≥ 90 g/L; 9. (2) Serum creatinine ≤ 1.5 × upper limit of normal (ULN), or glomerular filtration rate ≥ 60 mL/min calculated by CKD-EPI formula; 10. (3) AST and ALT ≤ 2.5 times ULN, or AST and ALT ≤ 5 times ULN in patients with a documented history of liver metastases; 11. (4) Serum total bilirubin ≤ 1.5 times ULN (total bilirubin must be < 3 times ULN for patients with Gilbert's syndrome); 12. (5) International normalized ratio (INR) ≤ 1.5 or prothrombin time ≤ 1.5 times ULN; 13. (6) Partial thromboplastin time (APTT) ≤ 1.5 × ULN; 14. 8. Females of childbearing potential must agree to practice highly effective contraception for the duration of the study and for ≥ 120 days after dosing and have a negative serum pregnancy test ≤ 7 days before the first dose of study drug. 15. 9. Nonsterilized men must agree to use highly effective contraception for the duration of the study and for ≥ 120 days after study drug administration. 16. 10. Life expectancy greater than 3 months.   **Cohort A Specific Inclusion Criteria:**   1. 1. Histologically confirmed Stage IIB-IIIA NSCLC (as defined by the American Joint Committee on Cancer, 8th edition). 2. 2. Confirmed eligibility for R0 resection for curative intent by thoracic surgeon assessment. 3. 3. Adequate cardiopulmonary function, confirmed to meet the requirement for surgical resection for curative intent.   **Cohort B Specific Inclusion Criteria:**  1. Histologically or cytologically confirmed locally advanced (Stage IIIA-IIIC), or metastatic (Stage IV) NSCLC not amenable to curative surgery or radiotherapy. |
| **Exclusion criteria** | 1. Patients with EGFR mutation, ALK gene rearrangement or ROS1 gene rearrangement:  (1) For patients with non-squamous cell carcinoma, if EGFR mutation status is unknown, tissue samples should be provided for local or central laboratory testing before enrollment;  (2) For patients with squamous cell carcinoma, if EGFR mutation status is unknown, it is not required to conduct test at screening;  (3) Testing at screening is not required if ALK gene rearrangement or ROS1 gene rearrangement status is unknown.  2. Allergic to any study drug or excipients.  3. Patients who have been treated with immune checkpoint inhibitors such as anti-PD-1, PD-L1 or CTLA-4 therapy.  4. Cohort A: patients who have received systemic platinum-based doublet chemotherapy; Cohort B: patients who have received systemic platinum-based doublet chemotherapy as advanced systemic therapy.  5. Patients received other approved systemic anticancer therapy or systemic immunomodulators 4 weeks before the first dose.  6. Cohort B: patients with refractory pleural effusion or ascites, such as pleural effusion or ascites requiring puncture and drainage 2 before the first dose.  7. Cohort B: Patients with active leptomeningeal disease or brain metastasis, such as central nervous system symptoms, requiring interventional therapy (including but not limited to radiotherapy, intracranial pressure lowering therapy, etc.).  8. Patients with any disease requiring systemic treatment with corticosteroids (daily dose of prednisone or equivalent > 10 mg) or other immunosuppressive drugs 14 days before grouping.  9. Active autoimmune disease or history of autoimmune disease that may recur.  10. History of interstitial lung disease, pneumonitis or uncontrolled systemic diseases, including diabetes, hypertension, pulmonary fibrosis, acute lung disease;  11. Serious infection occurred before grouping, including but not limited to hospitalization due to infectious complications, bacteremia or severe pneumonia; severe chronic or active infection (including pulmonary tuberculosis infection, etc.) requiring systemic (oral or intravenous) antibiotics within 14 days before grouping.  12. HBV deoxyribonucleic acid (DNA) must be < 500 IU/mL (or 2500 copies/mL) in inactive/asymptomatic carriers, patients with chronic or active hepatitis B virus (HBV) at screening.  13. Any major surgery requiring general anesthesia ≤ 28 before the first dose.  14. Presence of underlying medical conditions or alcohol/drug abuse or dependence that would impair the administration of the study drug, or that could affect the interpretation of the results, or result in a high risk of treatment complications.  15. Simultaneous participation in another therapeutic clinical study.  16. Pregnant or lactating women, or male and female patients planning to have children during the study.  17. Other conditions that the investigators consider inappropriate for participation in this trial, such as poor compliance. |
| **Sample Collection** | **Blood samples:**  Collect 5 mL of blood into an EDTA tube before administering the drug, centrifuged for 10 min at 4000 rpm to obtain fresh plasma before storing at -80°C until analysis. The sample must be processed within 2 hours.  **Fecal samples:**  Collect posterior feces after urination using plastic emesis basin and 15 mL sterile stool collector containing Swab DNA locker. Samples are allowed to be kept at room temperature temporally and brought to the laboratory within 24h after collection, where all samples should be homogenized and finally stored at -80°C.  **Sample transportation:**  (1) Select a thick-walled foam box to avoid breakage and sample loss during transport. Seal the foam box and place it inside an outer cardboard box and seal it with tape as well.  (2) Select an appropriately sized foam box according to the amount of dry ice. Place dry ice (10-15 kg required) and the samples (packed in plastic bags or disposable gloves) inside the box. Fill any empty space with crumpled newspaper to prevent leakage after the dry ice sublimates. |
| **Statistical**  **Methods** | **Sample size:**  Since this is an exploratory study, no inferential statistical tests are proposed in this trial, sample size is determined by the pragmatics of recruitment, such as patient flow and budgetary constraints.  **Statistical analysis of the population:**  1. Full Analysis Set (FAS): it includes all enrolled subjects who have received at least one dose of study drug according to the intention-to-treat (ITT) principle.  2. Efficacy Evaluable Analysis Set (EAS): it includes all subjects who have received at least one dose of study drug after enrollment and have undergone pathological assessment after surgical treatment.  3. Safety Analysis Set (SAS): it includes all enrolled subjects who have received at least one dose of study drug. The SS will be used for all safety analyses.  **Baseline statistical analysis:**  Baseline, demographic characteristics, baseline tumor characteristics, medical history, comorbid medications, vital signs, and termination of the trial will be summarized for all enrolled subjects. For continuous data, the mean, standard deviation, range of distribution and median will be calculated; absolute values, frequencies and percentages will also be calculated.  **Validity analysis:**  For efficacy endpoints in the dichotomous categories (including MPR, pCR and resectable rate), the number of cases and percentage of subjects under each classification will be summarized. For efficacy endpoints of the time-to-event type (like DFS), the number and percentage of subjects with events and censored will be summarized and plotted by the Kaplan-Meier curve.  **Security Analysis:**  All adverse events that occur during treatment will be summarized, including: (1) treatment-emergent adverse events (TEAEs) grade ≥ 3, (2) TEAEs leading to treatment termination or dose interruption/reduction/delay, (3) immune-related adverse events (IRAEs), (4) serious adverse events (SAEs), (5) deaths, (6) treatment-related adverse events (TRAEs). |
